# Stratospheric ozone, UV radiation, and climate interactions

**DOI:** 10.1007/s43630-023-00371-y

**Published:** 2023-04-21

**Authors:** G. H. Bernhard, A. F. Bais, P. J. Aucamp, A. R. Klekociuk, J. B. Liley, R. L. McKenzie

**Affiliations:** 1 Biospherical Instruments Inc, San Diego, CA USA; 2 Laboratory of Atmospheric Physics, Department of Physics, Aristotle University, Thessaloniki, Greece; 3 Ptersa Environmental Consultants, Pretoria, South Africa; 4 Antarctic Climate Program, Australian Antarctic Division, Kingston, Australia; 5 National Institute of Water & Atmospheric Research, Lauder, New Zealand

## Abstract

This assessment provides a comprehensive update of the effects of changes in stratospheric ozone and other factors (aerosols, surface reflectivity, solar activity, and climate) on the intensity of ultraviolet (UV) radiation at the Earth’s surface. The assessment is performed in the context of the Montreal Protocol on Substances that Deplete the Ozone Layer and its Amendments and Adjustments. Changes in UV radiation at low- and mid-latitudes (0–60°) during the last 25 years have generally been small (e.g., typically less than 4% per decade, increasing at some sites and decreasing at others) and were mostly driven by changes in cloud cover and atmospheric aerosol content, caused partly by climate change and partly by measures to control tropospheric pollution. Without the Montreal Protocol, erythemal (sunburning) UV irradiance at northern and southern latitudes of less than 50° would have increased by 10–20% between 1996 and 2020. For southern latitudes exceeding 50°, the UV Index (UVI) would have surged by between 25% (year-round at the southern tip of South America) and more than 100% (South Pole in spring). Variability of erythemal irradiance in Antarctica was very large during the last four years. In spring 2019, erythemal UV radiation was at the minimum of the historical (1991–2018) range at the South Pole, while near record-high values were observed in spring 2020, which were up to 80% above the historical mean. In the Arctic, some of the highest erythemal irradiances on record were measured in March and April 2020. For example in March 2020, the monthly average UVI over a site in the Canadian Arctic was up to 70% higher than the historical (2005–2019) average, often exceeding this mean by three standard deviations. Under the presumption that all countries will adhere to the Montreal Protocol in the future and that atmospheric aerosol concentrations remain constant, erythemal irradiance at mid-latitudes (30–60°) is projected to decrease between 2015 and 2090 by 2–5% in the north and by 4–6% in the south due to recovering ozone. Changes projected for the tropics are ≤ 3%. However, in industrial regions that are currently affected by air pollution, UV radiation will increase as measures to reduce air pollutants will gradually restore UV radiation intensities to those of a cleaner atmosphere. Since most substances controlled by the Montreal Protocol are also greenhouse gases, the phase-out of these substances may have avoided warming by 0.5–1.0 °C over mid-latitude regions of the continents, and by more than 1.0 °C in the Arctic; however, the uncertainty of these calculations is large. We also assess the effects of changes in stratospheric ozone on climate, focusing on the poleward shift of climate zones, and discuss the role of the small Antarctic ozone hole in 2019 on the devastating “Black Summer” fires in Australia. Additional topics include the assessment of advances in measuring and modeling of UV radiation; methods for determining personal UV exposure; the effect of solar radiation management (stratospheric aerosol injections) on UV radiation relevant for plants; and possible revisions to the vitamin D action spectrum, which describes the wavelength dependence of the synthesis of previtamin D_3_ in human skin upon exposure to UV radiation.

**Graphical abstract:**

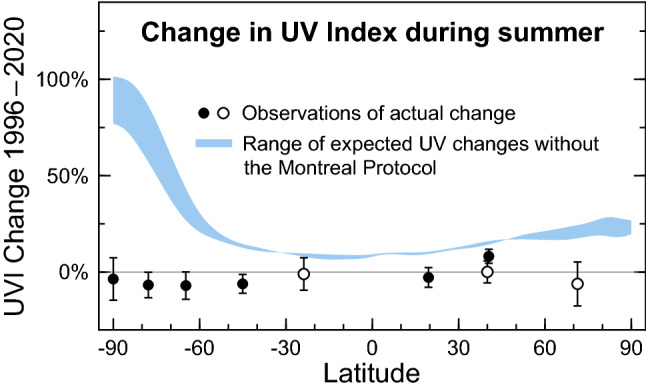

## Introduction

This Perspective is the first in a series of assessments^1^ prepared by members of the Environmental Effects Assessment Panel (EEAP) of the Montreal Protocol under the United Nations Environment Programme (UNEP). It focuses on the effects of changes in the ozone layer on climate and ultraviolet (UV) radiation at the Earth’s surface, the interactions between UV radiation and climate, and on the influence of other geophysical parameters affecting UV radiation. This assessment sets the stage for subsequent assessments in this series that address the consequences of the interconnected effects of stratospheric ozone depletion, UV radiation, and climate change on human health [1] (including the COVID-19 pandemic [2]), terrestrial [3] and aquatic [4] ecosystems, the carbon cycle [3, 4], air quality [5], natural and synthetic materials [6], and the fate of environmental plastic debris [7]. These assessments focus on new scientific knowledge that has accumulated since our last comprehensive assessment (*Photochem. Photobiol. Sci*., 2019, 18, 595–828) and up to August 2022. Many of these effects are assessed in terms of the benefits for life on Earth resulting from the implementation of the Montreal Protocol on Substances that Deplete the Ozone Layer [8] and its Amendments and Adjustments (henceforth “the Montreal Protocol”). These benefits were achieved by curbing depletion of stratospheric ozone, thereby limiting increases of UV radiation, and mitigating climate change. Further topics include assessments of observed trends in UV radiation, projections of UV radiation into the future, and advances in the monitoring and modeling of UV radiation.

## State of the science in 2018

The previous comprehensive assessment of the EEAP [9], which was based on the state of knowledge in 2018, concluded that the Montreal Protocol was highly beneficial for protecting the stratospheric ozone layer and limiting the rise of solar UV-B (280–315 nm) radiation at the Earth’s surface. Therefore, increases in erythemal (sunburning) UV radiation between the late 1970s (at the onset of anthropogenically induced stratospheric ozone depletion) and 2018 were negligible in the tropics, small (< 10%) at mid-latitudes (30–60°), and large (> 50%) only in polar regions.^2^ Furthermore, the implementation of the Montreal Protocol^3^ prevented increases in UV-B radiation since the mid-1990s. As a result, observed changes in UV radiation at mid-latitudes during the last ~ 3 decades were mainly controlled by clouds and aerosols instead of changes in stratospheric ozone. Statistically significant decreases in UV-B radiation consistent with ozone recovery had not yet been detected at mid- and low latitudes at the time of the previous assessment because of the large variability in UV-B radiation caused by factors other than ozone. Conversely, continuing decreases in clouds and aerosols (rather than changes in ozone) observed since the mid-1990s led to positive trends of UV radiation at several sites between 30° and 60° N. Several independent satellite records indicated that changes in large-scale patterns of clouds occurred between the 1980s and 2000s with consequences on UV radiation at the Earth’s surface.

In contrast to the tropics and mid-latitudes, variability of UV-B radiation in Antarctica remained very large, with near record-high erythemal UV radiation observed at the South Pole in spring 2015 and well below average values in spring 2016. The Arctic remained vulnerable to large decreases in total column ozone^4^ (TCO) and concomitant increases in UV-B irradiance whenever meteorological conditions led to a cold lower stratosphere in late winter and early spring. For example, greatly reduced stratospheric ozone concentrations during the second half of February 2016 led to increases of erythemal UV radiation of up to 60% above the climatological average over northern Scandinavia and northern Siberia.

By preventing the further growth of the Antarctic ozone hole, the Montreal Protocol also helped to reduce its effects on atmospheric circulation, which include shifts of climate zones in the Southern Hemisphere and associated changes in weather patterns. For example, changes in tropospheric circulation contributed to a decrease in summer temperatures over south-east and south-central Australia, and inland areas of the southern tip of Africa. Anomalously high TCO in the spring were significantly correlated with hotter-than-normal summers over large regions of the Southern Hemisphere and vice versa.

With the predicted recovery of stratospheric ozone over the next several decades, UV-B radiation was expected to decrease at all latitudes outside the tropics, with the greatest decreases predicted over Antarctica. A projection of the erythemal irradiance^5^ (quantified in terms of the UV Index^6^ or UVI) for the end of the twenty-first century (average of 2085 − 2095) relative to the current decade (average of 2010 − 2020) suggested that ozone recovery will lead to a decrease in the UVI by about 30% over Antarctica, and up to 6% over mid-latitudes. These projections were uncertain because future concentrations of stratospheric ozone will depend not only on the decrease of ozone-depleting substances (ODSs) controlled by the Montreal Protocol but also on the trajectory of concentrations of other greenhouse gases such as carbon dioxide and methane, which will greatly depend on policy decisions implemented in the coming decades. Changes in cloudiness were projected to result in small (up to 4%) localized increases in UVI over the mid-latitudes and tropics, and to decreases exceeding 10% in the Arctic. Reductions in reflectivity due to melting of snow and sea ice as well as shifting of the melting season were predicted to decrease above-surface UVI by up to 10% in the Arctic and by 2–3% around Antarctica. However, the increasingly ice-free Arctic Ocean and reductions in snow cover would lead to increases in UV radiation penetrating the water column and reaching land surfaces formerly covered by snow. Decreases in concentrations of aerosols over urban areas of the Northern Hemisphere were projected to increase the UVI by typically 5–10% and by up to 30% over heavily industrialized regions (e.g., southern and eastern Asia) as measures to control air pollution start to reduce contamination from aerosols towards pre-industrial levels. The extent of these changes was again determined to be greatly contingent on policy decisions.

## Current and future status of atmospheric ozone

Changes in atmospheric ozone concentrations in general and TCO in particular are regularly being assessed by the Scientific Assessment Panel (SAP) of the Montreal Protocol in coordination with the World Meteorological Organization (WMO) and UNEP. The information provided in this section is largely based on the SAP’s latest assessment [11] and provides the background for our assessment of the various effects resulting from changes in the ozone layer. We note that trends in TCO assessed by the SAP and summarized here refer to trends resulting mainly from human activities. The effects of natural cycles and events that affect TCO have been removed as part of the trend analysis. Such cycles and events include the solar cycle; the quasi-biennial oscillation (QBO; a pattern of alternating zonal winds in the tropical stratosphere); the El Niño-Southern Oscillation (ENSO; a pattern of alternating warm and cold sea surface temperatures of the tropical Pacific Ocean); the Arctic Oscillation (AO) and the Antarctic Oscillation (AAO), which both describe the back-and-forth shifting of atmospheric pressure between the poles and the mid-latitudes; the Brewer–Dobson circulation (a global-scale meridional circulation in the stratosphere); and aerosols from major volcanic eruptions [12].

### Changes in total column ozone outside the polar regions

**Fig. 1:**
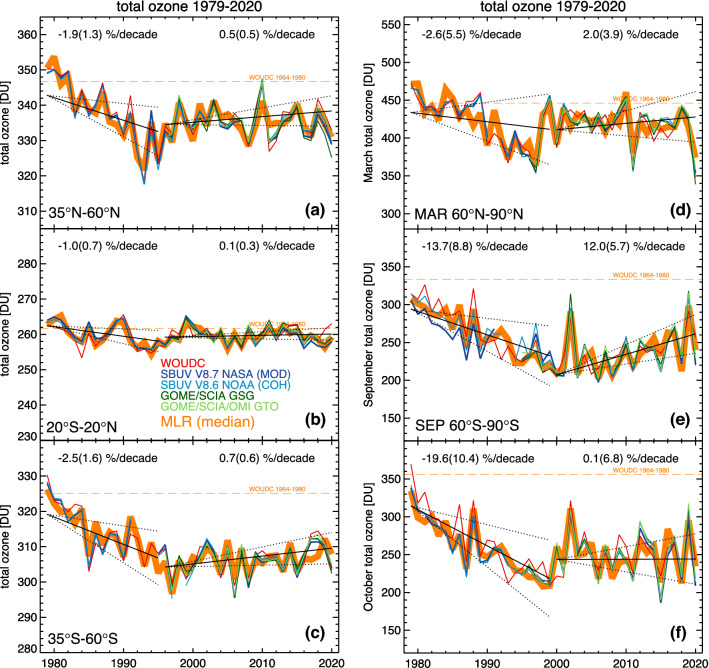
Time series of annual-mean TCO for the latitude bands **a** 35° N − 60° N, **b** 20° S − 20° N, and **c** 35° S − 60° S; and monthly mean TCO for **d** March in the Arctic (60° N − 90° N), **e** September in the Antarctic (60° S − 90° S), and **f** October in the Antarctic (60° S − 90° S). Colors indicate different ground- and satellite-based datasets. These are identified in the legend of panel (**b**) and defined as follows: *WOUDC* ground-based measurements from the World Ozone and UV data center (https://woudc.org/); SBUV V8.7 NASA (MOD): NASA Merged Ozone Data from the series of space-borne Solar Backscatter Ultraviolet (SBUV) instruments; SBUV V8.6 NOAA (COH): the NOAA cohesive dataset from several satellite sensors; GOME/SCIA GSG: the merged dataset from the space-borne Global Ozone Monitoring Experiment (GOME), the SCanning Imaging Absorption spectroMeter for Atmospheric CHartographY (SCIAMACHY), GOME-2A, and GOME-2B; and GOME/SCIA/OMI GTO: the merged data set from GOME, SCIAMACHY, the Ozone Monitoring Instrument (OMI), GOME-2A, GOME-2B, and TROPOspheric Monitoring Instrument (TROPOMI). The MLR (heavy orange line) dataset is the median of the five datasets described above and represents the input to the regression model applied by Weber et al. [12]. Solid black lines indicate linear trends calculated with this regression model before and after the peak in ODSs in 1996, respectively, and dotted lines indicate the two standard deviation (2σ) uncertainty of the estimated trends. Trend numbers are indicated for the pre (1979–1995) and post (1996–2020) ODS peak period in the top part of the plot. Numbers in parentheses are the 2σ trend uncertainty. The dashed orange line shows the mean TCO from 1964 until 1980 from the WOUDC data. Note that the scales of the ordinates are different in the six panels. Adapted from Weber et al. [12]

Signs of the ozone layer’s recovery outside the polar regions are now more robust compared to the SAP’s previous assessment [13] owing to updated trend models and additional four years of data. For the first time, small but statistically significant increases in TCO (of 0.4 ± 0.2% per decade) for the period 1996–2020 are now evident for the latitude band 60° S–60° N [12]. However, this positive trend is mostly driven by TCO changes in the Southern Hemisphere (Fig. 1). In the tropics (20° S–20° N) and northern mid-latitudes (35°–60° N), increases in TCO since 1996 have not been observed with certainty (Fig. 1a, b), and statistically significant trends (of 0.7 ± 0.6% per decade) have only been found for the southern mid-latitudes (35°–60° S) (Fig. 1c). Even though the Montreal Protocol entered into force more than 30 years ago, it was expected that the recovery of the ozone layer at mid-latitudes would only now start to become evident because the removal rate of ODSs controlled by the Montreal Protocol from the stratosphere is three to four times slower than the rate at which they were added [14]. Furthermore, year-to-year variability in TCO obscures the attribution of trends to declining concentrations of ODSs. Detecting significant increases in TCO outside Antarctica therefore requires much more time than the detection of its previous decline. In the upper stratosphere, however, the rate of increase in the ozone concentrations is larger, ranging between 1.5 and 2.2% per decade over the mid-latitudes of both hemispheres, and between 1 and 1.5% per decade in the tropics [11]. Since ozone column amounts in the upper stratosphere (above 32 km) are relatively small (typically less than 25% of the TCO at mid-latitudes), these increases contribute only modestly to the growth of TCO. Over the mid-latitudes, the present-day TCO (2018–2020 average) is still below the average of the period 1964–1980 by ~ 4% in the Northern Hemisphere and by ~ 5% in the Southern Hemisphere [11]. Reasons for these latitude-dependent changes in TCO are discussed in SAP’s 2022 assessment [11].

### Changes in total column ozone over Antarctica

Several studies have provided evidence that the Antarctic ozone hole is starting to recover [15–21]. Signs of recovery are strongest for the month of September, which is the key month for chemical destruction of ozone. Both ground-based and satellite data indicate a statistically significant positive trend in TCO of 12% per decade in September since 2000 (Fig. 1e). These increases are consistent with the decrease in the concentration of ODSs controlled by the Montreal Protocol [20]. However, there are still no significant trends for October (Fig. 1f) or later months because TCO in late spring is less sensitive to decreasing ODSs in the stratosphere compared to September. In a typical Antarctic winter, ozone is almost completely destroyed in the lower stratosphere by the end of September, which may explain why no recovery has yet been observed in October over the polar cap [12]. In addition, year-to-year variability is also larger later in the year [11].

Assuming continued adherence to the Montreal Protocol, concentrations of ODSs are projected to decline further, eventually resulting in the disappearance of the annually recurring ozone hole in the second half of the twenty-first century [11]. Until that time, large year-to-year variations in various ozone hole metrics are expected because of the sensitivity of chemical ozone destruction to temperature in the lower stratosphere in the presence of ODSs. Especially during the last few years, the depth and size of the Antarctic ozone hole have exhibited particularly large variability:

- In September and October 2019, the Antarctic ozone hole was the smallest on record since the early 1980s due to abnormally strong planetary wave^7^ activity originating in the subtropical Pacific Ocean east of Australia and over the eastern South Pacific [22–24]. These waves weakened the stratospheric polar vortex, which led to a warming of the polar stratosphere, starting in mid-August [25]. The resulting above-normal temperature in the lower stratosphere reduced the occurrence of polar stratospheric clouds (PSCs), which provide the surfaces for heterogeneous^8^ chemical reactions involving chlorine that result in catalytic destruction of ozone. The volume of PSCs dropped to almost zero by mid-September and the chemical processes leading to ozone depletion were therefore suppressed far earlier than usual. The average TCO over the polar cap (60°–90° S) in September and October 2019 was the highest over the last 40 years, and the minimum TCO for September 2019 was the highest since 1988. For the months of September, October, and November, the polar cap average TCO was higher by 29%, 28%, and 26%, respectively, compared to the mean of the 2008–2018 period [26].
- In contrast, the Antarctic ozone holes in spring 2020 and 2021 were amongst the largest and longest-lived in the observational record [27, 28]. These long-lasting ozone holes, extending to times when snow has melted, may have had impacts on Antarctic organisms [29]. Yook et al. [28] provided evidence that injection of smoke originating from the Australian “Black Summer” wildfires of early 2020 (Sect 5.1.2) may have contributed to the large ozone hole of 2020, while aerosols from the eruption of La Soufrière (13° N) on Saint Vincent in April 2021 may have played a role in the large ozone hole of 2021. (Aerosols injected into the tropical stratosphere disperse rapidly to high latitudes [30].) Furthermore, the lack of planetary waves during both years resulted in a cold and stable stratospheric vortex over Antarctica, which created conditions favorable for persistent ozone depletion [11, 20, 31]. Additionally, loss of ozone in early spring 2020 enhanced the strength and persistence of the vortex later in that year [32]. Even though large ozone holes will likely continue to occur in the future, either through dynamical variability alone, or exacerbated by large volcanic eruptions or major inputs of smoke into the stratosphere, the recovery of the ozone hole is expected to continue [27].

The large year-to-year variability in the TCO observed thus far resulted in large year-to-year variations in UV radiation in Antarctica (Sect. 7.1.1). For example, the UVIs measured at the South Pole in 2019 were some of the lowest since the start of measurements in 1991, while those in 2020 set new record highs. The recovery of the Antarctic ozone hole is generally more difficult to detect with UV-B radiation than ozone data because signs of recovery are most pronounced in September [15, 33] when the UVI in Antarctica is still very low. Factors other than ozone that affect UV radiation (Sect. 6) lead to additional variability, hampering detection of recovery further.

**Fig. 2:**
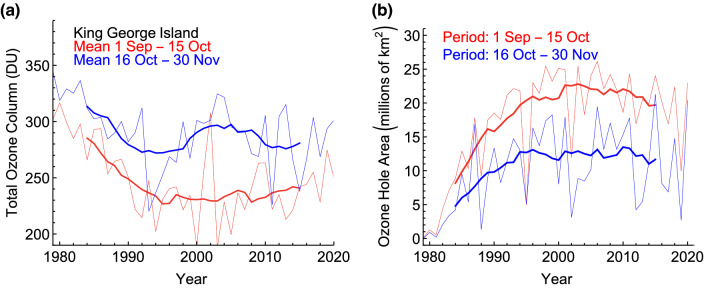
**a** Time series of TCO at King George Island (62° S), averaged from 1 September to 15 October (red line) and from 16 October to 30 November (blue line). **b **Evolution of the ozone hole area averaged from 1 September to 15 October (red line) and from 16 October to 30 November (blue line). Bold lines indicate 11-year centered moving averages calculated from annual data. Adapted from Cordero et al. [34]

Using observations from satellites between 1978 and 2020, a recent study [34] compared annual averages of the depth and area of the Antarctic ozone hole for early spring (1 September–15 October) and late spring (16 October–30 November). This analysis is of high relevance for assessing trends in UV radiation over Antarctica because UV radiation is generally much greater later in spring when the Sun is higher in the sky even though TCO is typically much lower earlier in spring. Figure 2a shows TCO averaged from 1 September to 15 October (red line) and from 16 October to 30 November (blue line) at King George Island (62° S), located near the northern tip of the Antarctic Peninsula. For the earlier period, the 11-year moving average of TCO was lowest around the year 2000, when the concentration of ozone-depleting chlorine and bromine compounds in the stratosphere was close to its maximum, and average TCO appears to be increasing since this time. The observation at this station is consistent with the positive trend in Antarctic TCO for September shown in Fig. 1e. Conversely, and consistent with Fig. 1f, there is no clear indication that TCO is also recovering in the later period. Similarly, the size of the ozone hole—quantified as the area with TCO below 220 Dobson Units (DU)—appears to be decreasing faster in early spring (Fig. 2b).

### Changes in total column ozone over the Arctic

While there is still no clear evidence of ozone recovery in the Arctic, it is expected that signs of recovery would first be detected in March because chemical ozone loss in the Arctic is typically largest in this month [35].

Figure 1d indicates that TCO in March averaged over the northern polar cap (63°–90° N) is indeed increasing by 2% per decade, but this small positive trend is not statistically significant because of the large interannual dynamical variability observed for this latitude belt [11].

Sporadic ozone depletion events continue to occur in the Arctic. An exceptionally large episode of stratospheric ozone depletion was observed in late winter and early spring (February–April) of 2020 [36], exceeding in severity the previously reported event of 2011 [37]. The TCO averaged over 63°–90° N for this 3-month period was 340 Dobson Units (DU), which is 100 DU below the mean of the period 1979–2019 and the lowest since the start of satellite measurements in 1979. These low values of TCO in 2020 were partially caused by a strong and long-lived polar vortex, which provided ideal conditions for chemical ozone destruction to take place. Temperatures low enough to form PSCs within the vortex developed early in the season, and on average enclosed about a third of the vortex volume [35, 36, 38–41]. Furthermore, the strong vortex also inhibited replenishment of Arctic ozone from lower latitudes [11]. These conditions are unique in the ~ 40 years of measurements, making 2020 the year with the largest loss of Arctic ozone on record. The large ozone hole observed over Antarctica six months later is a coincidence and cannot be attributed to a known common cause.

The unprecedented depletion of Arctic ozone in winter/spring of 2019/2020 contrasts with the conditions in the boreal winters of 2018/2019 and 2020/2021. In both winters, major stratospheric warmings occurred in January [42–44], which limited overall ozone loss. As a result, the minimum TCO in March 2019 (defined as the minimum of the daily mean TCO within an area that encloses the Arctic polar vortex and is surrounded by the 63° N contour of “equivalent latitude” [45]) was the highest since 1988 [46], and the minimum TCO in March 2021 was identical to its average value since the start of satellite observations in 1979 [47]. Such large year-to-year variations in Arctic ozone depletion, which are driven by differences in meteorological conditions, are expected to continue for as long as concentrations of ODSs remain elevated [11, 41, 48]. Furthermore, winters with a warm stratosphere (and little ozone depletion) will likely randomly alternate with winters with a cold stratosphere (and large ozone depletion). A recent study [49] provides evidence that years with a cold stratospheric Arctic vortex are getting colder. Reduced stratospheric temperatures will likely result in more PSC formation and lead to more chemical ozone loss via catalytic processes. As a consequence, ozone-depletion events as large or even larger than the one observed in 2020 [e.g., 36] will likely re-occur throughout the twenty-first century until concentrations of ODSs have substantially decreased. The magnitude of stratospheric cooling in the future will critically depend on the development of greenhouse gas (GHG) concentrations and on variability in the amount of water (H_2_O) vapor in the stratosphere [11, 49]. Under the scenario with the highest concentration of GHGs and H_2_O, sporadic springtime increases in UV radiation in the Arctic could be somewhat larger at the end of the twenty-first century than those observed in 2020 [49].

### Effects of greenhouse gases on stratospheric ozone

This section briefly discusses the effects of changes in the atmospheric concentration of GHGs that are responsible for global warming but are also relevant to stratospheric ozone changes. The SAP’s latest report [11] discusses these processes in more detail. Increases in GHGs affect ozone depletion in several key ways [50]. First, radiative cooling of the *polar* stratosphere (promoted by GHGs during winter months) enhances the formation of PSCs. These clouds provide the surfaces for heterogeneous chemical reactions that lead to the destruction of ozone, thereby *decreasing* ozone concentrations. Second, cooling of the upper stratosphere at *extrapolar* latitudes reduces the rates of gas-phase chemical reactions that lead to ozone loss, thereby *increasing* ozone concentrations in the upper stratosphere. Third, changes in the concentrations of nitrous oxide (N_2_O) and methane (CH_4_), which are both GHGs, also affect ozone concentrations chemically because both gases are also key sources of reactive species in catalytic cycles (the NOx and HOx cycles, respectively) that destroy ozone. The NOx cycle dominates in the middle stratosphere (approximately 25–35 km) while the HOx cycle is mostly contributing in the lower stratosphere. Fourth, increases in GHG concentrations are expected to strengthen the Brewer–Dobson circulation, which describes the redistribution of ozone from tropical to extratropical regions [51]. Fifth, global warming induced by increases in GHGs increases the flux of “very short-lived substances” (VSLS) into the stratosphere as further explained in the following.

VSLS are ozone-depleting halogen-containing substances with a lifetime of less than six months that are mostly produced by natural processes, for example, by macroalgae (seaweed) and phytoplankton. About 25% of bromine entering the stratosphere in 2016 was from VSLS [13], with the majority originating from oceanic sources. While stratospheric bromine is a relatively minor constituent by volume, it is an important contributor to ozone depletion. Per atom, bromine is about 60–75 times (depending on the concentration of GHGs) more effective in destroying ozone than is chlorine [52]. A recent modeling study [53] examined the effect of climate change on changes in bromine from oceanic sources. The study assumed the Representative Concentration Pathway^9^ RCP 6.0 GHG scenario and concluded that the flux of brominated VSLS compounds from the ocean to the atmosphere will increase by about 10% over the twenty-first century for all latitudes with the exception of the Arctic. The increase will be even greater over the Arctic because of the projected decrease in sea ice, which is currently hindering the escape of brominated compounds from the ocean. By the end of the twenty-first century, almost the entire polar ocean will likely be exposed in August and September and sea ice will no longer curtail ocean–atmosphere fluxes of brominated compounds. This study is one example of an indirect effect of climate change on the concentration of substances that promote stratospheric ozone depletion.

### Estimates of total column ozone during the twenty-first century

Projections of TCO into the future are available from chemistry-climate models (CCMs), which were run for different future emissions scenarios as part of a coordinated, multi-model activity where all models follow the same protocols to perform a comparable set of simulations [11, 54]. Uncertainties associated with these projections arise mainly from the assumed future trajectories of emissions of GHGs and pollutants. The models were run in the framework of CMIP6^10^ simulations and follow a new set of future emissions scenarios, the Shared Socioeconomic Pathways (SSP^11^) [55], which assume compliance with the Montreal Protocol and its Amendments. The ozone projections for the different SSPs are therefore based on the same evolution of controlled ODSs and depend only on the evolution of GHGs and other pollutants.

**Fig. 3:**
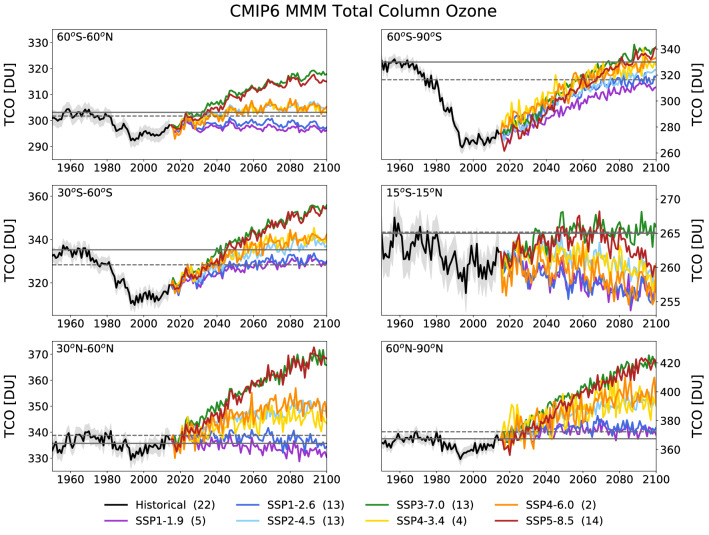
Regional average CMIP6 multi-model annual-mean TCO for the historical period (1950–2015) (black line), and the future (2015–2100) based on seven SSP scenarios (colored lines). The six panels show results for different latitudinal bands, indicated in the top left of each panel. The number of models participating in each simulation is shown in parentheses in the legend. The light gray envelope indicates the model spread for the historical simulations (calculated as the standard error). Total ozone columns for the 1960 and 1980 annual means are given by the solid and dashed horizontal gray lines, respectively. Note that the scales of the ordinates are different in the six panels. Reprinted from Keeble et al. [54]

The new simulations for the evolution of TCO towards the year 2100 support conclusions similar to those presented in a previous assessment of the SAP [13]. Figure 3 depicts the evolution of the annual-mean TCO averaged over different latitude bands for the period 1950–2100. The projections are based on a set of CMIP6 CCMs, which were run for the historical period 1950–2015 as well as for different scenarios for the future period 2015–2100. Year-to-year variability in these simulations is the result of internal variability (sometimes called “weather noise” [13]).

In summary, for scenarios with stabilizing or slightly decreasing concentrations of GHGs (SSP2-4.5, SSP4-3.4, and SSP4-6.0), the near-global mean (60° S–60° N) TCO is projected to return to historic levels (year 1980) by the middle of the twenty-first century (around year 2040) and remain at those levels until 2100. For scenarios with continued GHG increases (SSP3-7.0 and SSP5-8.5), the TCO is projected to return to 1980 levels sooner and significantly exceed historic levels throughout the latter half of the twenty-first century. This overshoot, which has also been termed “super-recovery”, results from the fact that increases in GHGs cool the upper stratosphere. This cooling reduces the rates of gas-phase chemical reactions that destroy ozone, and as a result, ozone concentrations increase. In contrast, and despite the assumption that halogenated ODSs will continue to decline throughout this century, TCO is not projected to return to historic levels by 2100 for scenarios with small GHG emissions (SSP1-1.9 and SSP1-2.6) and is projected to decrease in the tropics [11]. The consequences of these changes in TCO on UV radiation at the Earth’s surface, and its dependence on the GHG scenario, are discussed in Sect. 8.

## Benefits of the Montreal protocol

Benefits of the Montreal Protocol can be both direct (curbing stratospheric ozone depletion and limiting increases of UV radiation) and indirect (effects on climate). This section provides new information on both benefits.

### Direct effects of the Montreal protocol on stratospheric ozone depletion and UV radiation

The phase-out of ODSs mandated by the Montreal Protocol has already limited increases in UV radiation at the Earth’s surface. To demonstrate this beneficial effect, McKenzie et al. [56] compared seasonal means of the daily maximum UVI measured at the Earth’s surface with UVI data derived from results of two CCMs that assumed either the “World Avoided” scenario, where emissions of ODSs would have continued without regulation, or the “World Expected” scenario, where ODSs are curbed in compliance with the Montreal Protocol and its Amendments. The ground-based measurements were made at 17 mostly clean-air sites (latitude range 73° N–90° S) by state-of-the-art spectroradiometers. Trends in the UVI over 1996–2018 derived from measurements at sites with sufficiently long data records were found to be either small (< ± 10% per decade at Antarctic sites) or not significantly different from zero. These estimates matched calculations following the World Expected scenario within the limits of the measurement uncertainty. In contrast, without the Montreal Protocol, the UVI at northern and southern latitudes of less than 50° would have increased by 10–20% between the early 1990s and 2018. For southern latitudes exceeding 50°, UVI values would have surged by between 25% (year-round at the southern tip of South America) and more than 100% (South Pole in spring and summer).

**Fig. 4:**
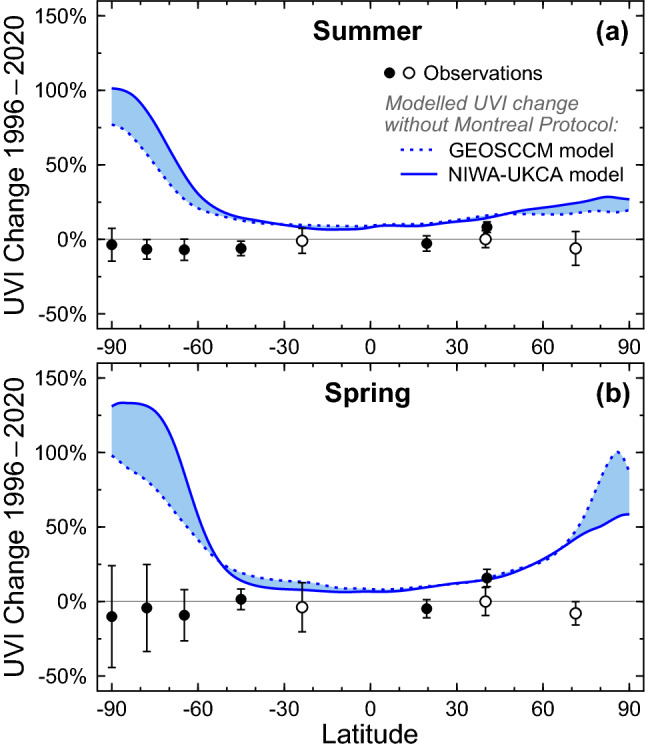
Comparison of relative changes in the UVI between 1996 and 2020 for **a** summer and **b **spring, derived from observations at nine ground stations (black symbols) and calculated from results of two chemistry-climate models (blue lines). Both climate models assume the “World Avoided” scenario where emissions of ozone-depleting substances are not controlled by the Montreal Protocol. Blue shading indicates the range of these model projections. Ground stations include South Pole (90° S), Arrival Heights (78° S), Palmer Station (65° S), Lauder (45° S), Alice Springs (24° S), Mauna Loa (20° N), Boulder (40° N), Thessaloniki (41° N), and Barrow (71° N). Ground stations with a near-complete data record for 1996–2020 are indicated by solid symbols. Sites with less than 24 years of data are shown with open symbols. Error bars indicate the 95% confidence interval of the regression model. Updated from McKenzie et al. [56]

Figure 4 shows an update of the work by McKenzie et al. [56] including also UVI measurements from 2019 and 2020, and focusing on sites with at least 15 years of observations between 1996 and 2020. With the exception of Thessaloniki (41° N), changes in the UVI over this time period have been smaller than ± 11% at all sites for both summer (Fig. 4a) and spring (Fig. 4b), and smaller than the “World Avoided” scenarios projected by the two CCMs (GEOSCCM^12^ [57] and NIWA-UKCA^13^ [58]), confirming that the Montreal Protocol has prevented large increases in UV radiation, in particular at southern latitudes higher than 60°. For example, without the Montreal Protocol (blue lines in Fig. 4), the UVI at the South Pole would by now have more than doubled in spring, while the ground-based measurements indicate a decrease of 10 ± 34% (± 2 standard deviations). Projected changes for high latitudes in the Northern Hemisphere are generally smaller because ozone depletion over the Arctic is less severe than that over the Antarctic (Sec 3.2 and 3.3). The relatively large increases in the measured UVI at Thessaloniki (16% for spring and 8% for summer) are mostly caused by reductions in atmospheric aerosols at this urban site resulting from air pollution control measures (Sect. 6.1) and are not the result of decreases in ozone.

### Indirect effects of the Montreal protocol on climate

Most ODSs controlled by the Montreal Protocol are also potent GHGs with Global Warming Potentials (GWPs) that are substantially larger than those of carbon dioxide (CO_2_) on a molecule-by-molecule basis. The climate forcing of halocarbons has greatly increased during the last century. For example, over the second half of the twentieth century, the combined direct radiative effect of all ODSs was the second largest contributor to global warming after CO_2,_ with approximately one third of the radiative forcing^14^ (RF) of CO_2_ [59]. The climate effects of ODSs were already anticipated during the establishment of the Montreal Protocol [60], and their impact on climate has been continuously revised since the ratification of the Montreal Protocol [13, 61, 62]. Work on assessing the contribution of ODSs to global warming has continued during the last four years; however, the net effect of ODSs on global temperatures is still highly uncertain [Chapters 6 and 7 of 63] because some of the warming that ODSs induce is offset by their effect on stratospheric ozone. Specifically, since ozone is also a GHG, depletion of ozone caused by ODSs has a cooling effect, but the magnitude of this effect (hereinafter termed “indirect forcing from ozone depletion”) is uncertain. On one hand, two single-model studies have reported a very large cancelation of the direct forcing by ODSs by the indirect forcing from ozone depletion of up to 80% [64, 65], and two multi-model studies using an “emergent constraint approach”^15^ based on CMIP6 models came to a similar conclusion [66, 67]. On the other hand, additional studies, which were part of several model intercomparison projects, concluded that the climatic effect from ODS-induced ozone depletion is either small or negligible [68–72]. According to Chiodo and Polvani [72], the four studies that have calculated a large effect on climate from ozone depletion have weaknesses (e.g., one study was based on a short time period, one study had a large ozone bias, and the remaining two studies assumed unrealistically strong ozone depletion), while the other studies that indicate a small indirect forcing from ozone depletion are more reliable because they are consistent with multi-model means of the CMIP5 and CMIP6 models, as summarized by Checa‐Garcia et al. [68]. However at this time, results from the two groups of studies cannot be reconciled. Because of these discrepancies, the latest (6th) report of the Intergovernmental Panel on Climate Change (IPCC) [specifically, Chapter 7 of 63] does not attempt to quantify the indirect forcing from ozone depletion, in contrast to previous IPCC reports [e.g., 73].

In the following, we summarize the results of recent studies that evaluate the amount of global warming that has been avoided due to the Montreal Protocol’s control of ODSs. All studies implicitly calculate the indirect forcing from ozone depletion and take this forcing into account when computing the net effect of the Montreal Protocol on surface temperatures. However, because of the uncertainty in calculating this feedback, the resulting effect on temperature is also uncertain. Still, taken together, these new studies further demonstrate the effectiveness of the Montreal Protocol in limiting temperature rise at the Earth’s surface.

Goyal et al. [74] used a coupled atmosphere–ocean–land–sea ice model to re-evaluate the Montreal Protocol’s effect on global warming from the control of ODSs. The study considered ODSs that have contributed substantially to stratospheric chlorine concentrations, namely the chlorofluorocarbons (CFCs) CFC-11 and CFC-12, as well as the CFC substitutes HCFC-22, HFC-125 and HFC-134a. Increases in GHG concentrations (including the concentrations of these ODSs) were described in this model by RCP 8.5, which leads to the strongest warming at the surface of the Earth. The study determined that, as of 2019, the Montreal Protocol has avoided warming between 0.5 to 1.0 °C over mid-latitude regions of Africa, North America, and Eurasia and as much as 1.1 °C warming in the Arctic. In addition to quantifying the benefits from the Montreal Protocol that have already been realized, Goyal et al. [74] also assessed the Montreal Protocol’s effect on the future climate for the RCP 8.5 scenario. Projected temperature increases that are likely to be averted by 2050 are in the order of 1.5 °C–2 °C over most extrapolar land areas, and between 3 °C and 4 °C over the Arctic. Averaged over the globe (including the oceans), about 1 °C warming would be avoided by 2050, which corresponds to about 25% mitigation of global warming expected from all GHGs.

A separate study [59] found that, over the period 1955–2005, ODSs were responsible for about one third of warming globally and about half of the warming in the Arctic. Since changes in Arctic temperatures have a direct effect on sea ice loss, Polvani et al. [59] concluded that ODSs contributed half of the forced Arctic sea ice loss in the latter half of the twentieth century. These results were recently confirmed [75], showing that Arctic warming and sea ice loss from ODSs are slightly more than half (52–59%) of those from CO_2_.

More recently, Chiodo and Polvani [72] calculated that stratospheric ozone depletion from ODSs only cancels about 25% of the RF from ODSs, in agreement with recent studies [e.g., 68]. The net RF of ODS is 0.24 W/m^2^ accordingly, which amounts to nearly one third of the RF of CO_2_ over the period 1955–2005, emphasizing the large RF effect of ODSs on tropospheric temperatures.

In summary, recent model calculations demonstrate a large effect of the Montreal Protocol in limiting global warming, but these results are subject to large uncertainties because the cooling effect resulting from ODS-induced ozone depletion is quantitatively not well reproduced by CCMs. The influence of ODSs on climate is an area of active research and it is expected that refinements to chemistry-climate models will further reduce uncertainties in estimating the effect of the Montreal Protocol on surface temperature.

In one of the latest Amendments of the Montreal Protocol (the 2016 Kigali Amendment [76]), the phase-down of hydrofluorocarbons (HFCs)—replacement chemicals of ODSs that do not harm the ozone layer but have a large GWP—is regulated. Without this amendment, the continued increase in atmospheric HFC concentrations would have contributed 0.28–0.44 °C to global surface warming by 2100. In contrast, the controls established by the Kigali Amendment are expected to limit surface warming from HFCs to about 0.04 °C in 2100 [77].

An unexpected slowdown in the decline of the atmospheric concentration of CFC-11 was observed after 2012 [78] and was partially caused by new emissions from eastern China (primarily the northeastern provinces of Shandong and Hebei). These emissions were likely due to new production and use [79]. They were initially of concern as they would delay recovery of ozone [80] and make a small but significant contribution to global warming [81]. The emissions appear to have been eliminated [82–84] and likely did not have a significant effect on dates of recovery of the ozone hole [85–88]. However, if similar emissions were to re-occur and last longer, effects on climate could be significant.

If the production of ODSs had not been controlled by the Montreal Protocol, biologically active UV-B radiation causing plant damage [89] could have increased by about a factor of five over the twenty-first century^16^ [90]. The ensuing harmful effects on plant growth were estimated to result in 325–690 billion tons less carbon held in plants by the end of this century. This reduction in carbon sequestration would have resulted in an additional 115–235 parts per million of CO_2_ in the atmosphere, causing an additional rise of global mean surface temperature of 0.5–1.0 °C. However, these estimates have large uncertainties and should be viewed with caution because the “generalized plant damage action spectrum” [89] used in the calculations does not account for the variety of plant responses across species and ecosystems. Furthermore, experiments (summarized by Ballaré et al. [91]) have not yet established whether the assumed sensitivity of plants to increases in UV-B radiation (i.e., a 3% reduction in biomass for every 10% increase in UV-B radiation for the “reference” scenario considered by Young et al. [90]) can be extrapolated to the very large increases in UV-B radiation simulated in this study. For example, Young et al. [90] did not consider that plants have protective mechanisms against damaging amounts of UV radiation, e.g., by synthesizing UV-absorbing compounds [e.g., 3, 92–95]. Such adaptation would mitigate the net CO_2_ flux into the atmosphere. Conversely, enhanced photodegradation of organic matter under elevated UV radiation would release additional CO_2_ into the atmosphere [96]. For more details, see Box 1 of Barnes et al. [3].

In conclusion, the studies assessed above provide further evidence that the Montreal Protocol is not only vital for the recovery of the ozone layer, but also for the reduction of global warming. The Montreal Protocol is, therefore, considered to be one of the most successful international treaties to date mitigating anthropogenic climate change.

## Effects of recent changes in stratospheric ozone on climate and weather

An in-depth assessment of the two-way interactions between changes in stratospheric ozone and climate is part of the SAP’s latest report [11]. Here, we focus on a subset of this assessment, and emerging topics. We also highlight the effects of Antarctic and Arctic ozone depletion on the climates of the Southern and Northern Hemisphere, respectively, and assess how these changes impact temperature and precipitation at the Earth’s surface as well as the extent of Antarctic sea ice and snow coverage.

### Effects of Antarctic ozone depletion on Southern Hemisphere climate

By enhancing cooling of the stratosphere, Antarctic ozone depletion has caused a poleward shift of climate zones and has been the primary driver of climate change in the Southern Hemisphere during summer in recent decades [97 and Sect. 5.1.1]. An influence of stratospheric ozone changes on sea surface temperature (SST) of the Southern Ocean may also be expected. However, current climate models have generally not been able to reliably reproduce observed changes in SST at high southern latitudes [98]. Recent modeling has provided evidence that changes in atmospheric ozone during the latter half of the twentieth century may be responsible for about one third of the observed warming in the upper 2000 m of the Southern Ocean (30°–60° S) [99]. About 60% of this contribution can be attributed to increases in tropospheric ozone—partly caused by increasing downward transport of ozone from the stratosphere to the troposphere and partly by enhanced production of ozone in the troposphere [100]—and the other 40% to stratospheric ozone depletion [99].

Antarctic sea ice cover increased between 1978 and 2015 [101, 102] and has subsequently shown a general decline with large year-to-year variability [103], which is still not completely understood (Sect. 5.1.3). Atmosphere–ocean interactions are intimately linked to the formation and dissipation of sea ice. However, the influence of ozone depletion on Antarctic sea ice is largely masked by other climate processes.

#### Shifting of climate zones

The effect of stratospheric ozone depletion on the summertime large-scale atmospheric circulation in the Southern Hemisphere has recently been confirmed and substantiated [97]. The primary effect has been the poleward shift of the tropospheric westerly winds over the Southern Ocean during the latter part of the twentieth century. The location of these tropospheric winds is quantified with the Southern Annular Mode (SAM^17^) index. The poleward shift of these winds has led to a more positive state of the SAM during summer [104–106]. This shift has affected regional temperature patterns [104] as well as precipitation in parts of Australia and South America [107], and Antarctica [105]. Specifically, stratospheric ozone depletion led to a tendency for more precipitation in parts of Australia, and less rain in South America. As an example, Yook et al. [28] provide evidence that the large Antarctic ozone holes of 2020 and 2021 (Sect. 3.2)—which were likely influenced by the Australian wildfires of early 2020 and the eruption of La Soufrière in April 2021, respectively—contributed to anomalously strong westerly winds over much of the Southern Ocean, anomalously cool conditions over the Antarctic plateau, anomalously warm conditions over the Antarctic peninsula, and anomalously cool conditions over much of Australia with flooding rains across the south-east of the continent. These anomalies are consistent with those observed in other years with large Antarctic ozone holes [105].

As a direct result of the Montreal Protocol, recovery of stratospheric ozone observed since the end of the twentieth century reversed cooling trends of the Southern Hemisphere’s lower stratosphere [21, 108]. However, warming trends observed post-2001 are about 50–75% smaller in magnitude than the cooling trends during the era of progressing ozone depletion. These changes in stratospheric temperature have also halted or partially reversed the poleward shift of climate zones [97].

Projections of the future climate for the Antarctic region under the 6th phase of the Coupled Model Intercomparison Project (CMIP6) [109] suggest that ozone recovery over the first half of the twenty-first century will tend to shift the westerly jet^18^ equatorward during summer. This would lead to a reversal of the changes in air and sea temperature at the surface—as well as in precipitation and in the zonal wind speed over Antarctica and the Southern Ocean—that were observed during the period of progressively worsening ozone depletion in the late twentieth century. However, this shift in the westerly jet is countered by the effects of both tropospheric warming and stratospheric cooling associated with increases in GHGs. The magnitude of this effect will depend on the GHG scenario defined by SSPs. Low-emissions scenarios (SSP1-2.6 and SSP2-4.5) tend to result in little overall change in the jet’s position, while high-emission scenarios (e.g., SSP5-8.5) tend to cause an overall poleward forcing, particularly outside of the summer season. In the second half of the twenty-first century, GHG effects dominate under all emissions scenarios, with the westerly jet strengthened and placed further poleward than before the ozone hole era. However, projections of how this shift will affect weather patterns at southern mid and high latitudes (including South America, South Africa and Australia) are subject to the strong dependence on GHG scenario and climate feedbacks (e.g., changes in sea ice and ocean temperatures), which may develop over the next 50 years, plus the limited ability of models to take all these processes into account on a regional scale.

#### Causes and consequences of the 2019/2020 “Black Summer” fires

A topic that has emerged since our previous assessment [9] is the role played by Antarctic ozone variability in recent extreme weather and climatic conditions, and the follow-on effects of these extremes for stratospheric ozone concentrations.

From mid-2019 to early 2020, a series of devastating wildfires occurred in Australia, particularly along parts of the eastern coast, affecting over 10 million hectares. The overall severity of these 2019/2020 “Black Summer” fires was exacerbated by exceptionally hot and dry weather conditions combined with rainfall deficits over several years. As shown by Lim et al. [110], anomalously hot and dry conditions in subtropical eastern Australia from austral spring to early summer are favored in years when the Antarctic stratospheric winter vortex is weak. Weak vortex conditions are promoted when planetary-scale (Rossby) waves disturb and warm the Antarctic atmosphere and reduce the overall amount of stratospheric ozone depletion in spring. The strong warming of the Antarctic stratosphere that occurred in September 2019 is a specific case of a weak vortex that has been linked with the 2019/2020 Black Summer fires [25, 111–117]. Specifically, downward coupling from the Antarctic stratosphere promoted a strong negative phase of the tropospheric SAM at mid-latitudes in summer, which reduced precipitation over Australia and further exacerbated fire conditions [115].

While the fires were mainly promoted by the weak polar vortex, the reduced ozone depletion resulting from the weak vortex may have been an exacerbating factor. This connection was studied by Jucker and Goyal [117] who found that surface conditions were influenced by anomalously high concentrations of ozone in the lower stratosphere that accompanied the stratospheric warming event and delayed the stratosphere–troposphere coupling. This suggests that ozone recovery could further promote a seasonal delay in stratosphere–troposphere coupling under weak vortex conditions. On the other hand, stratospheric warming events, such as that observed in 2019, appear to be less likely in a future climate [118] as increasing concentrations of GHGs will cool the stratosphere.

One consequence of the Black Summer fires was that superheated air from these fires produced large-scale pyrocumulonimbus clouds, which forced injection of an unprecedented amount of smoke and tropospheric air into the lower stratosphere [119–125]. From there, this air rose to heights of up to 35 km where it had persistent effects across a wide latitude band for several months [123, 126]. Ozone-poor tropospheric air in the rising plume reduced TCO by up to 100 DU locally [119, 123, 127], with impacts on UV radiation at the Earth’s surface. The rising air also increased mixing ratios of water vapor in the lower stratosphere at southern mid-latitudes [123, 127] where it may have depleted ozone through enhanced heterogeneous reactions [128], although the magnitude of this effect is unclear [129]. The plume also contained significant quantities of black carbon aerosol and reactive gases, which affect stratospheric chemistry [31, 130–132]. Quantifying the overall effect of the Black Summer fires on stratospheric ozone is still the subject of ongoing research. Additional information on the fire’s impact on stratospheric chemistry is provided in SAP’s latest assessment [11].

#### Effects on sea ice extent and snow coverage

The effects of ozone depletion on temperature and air circulation over Antarctica may also change snow and ice cover on the Antarctic continent and the extent of sea ice. For example, interactive climate models [97, 133], which are state-of-the-art in representing the complex interplay between effects of transport and dynamics [134], have demonstrated that ozone depletion has influenced near-surface winds over the Southern Ocean during summer and could, thus, potentially affect sea ice extent**.** However, as discussed below, these linkages are still not well understood. Changes in ice or snow coverage are important because they modify the reflectivity of the surface, which in turn changes downwelling UV radiation (Sect. 6.2).

The sea ice zone surrounding Antarctica shows strong seasonal variability [101, 102, 135]. There has been marked interannual variability during the last four decades, particularly in the last years, with regionally opposing patterns of change [Chapter 2 of 63]. Antarctic sea ice expanded between 1979 (the start of satellite measurements) and 2015, although only in the transitional seasons. Trends in both summer and winter were not significant. After this period of increase, the extent of Antarctic sea ice declined dramatically during the austral springs of 2016 and 2017 [101], reaching a record low on 1 March 2017, which was 27% below the mean of annual minima calculated for 1978–2016. However, a partial recovery was observed between 2017 and 2021 (https://climate.nasa.gov/ask-nasa-climate/2861/arctic-and-antarctic-sea-ice-how-are-they-different/). Several studies examined the reasons for this recovery [136–138]; however, none of these studies found robust evidence that trends or variations in stratospheric ozone contributed to this phenomenon.

As discussed in Sect. 5.1, the effect of stratospheric ozone depletion on temperatures at the surface of the Southern Hemisphere is primarily mediated by changes in the SAM during summer. Observational studies have shown that the seasonal response to trends in the SAM has resulted in the cooling of the SST around Antarctica in autumn, which should have promoted an overall increase in sea ice extent in that season, consistent with observations between 1979 and 2015 [139–141]. Furthermore, ozone depletion has been linked to a reduction in downwelling long-wave^19^ radiation. This reduction would also cool the Southern Ocean [142, 143]. In contrast to these studies, results from state-of-the-art earth-system models clearly indicate that ozone depletion in the second half of the twentieth century should have caused a reduction in sea ice extent, mainly by promoting the redistribution of ocean heat content [140, 141, 143–145]. However, only a subset of leading climate models can adequately capture the observed link between the SAM and autumn changes in sea ice [133]. In general, current climate models do not provide a consistent representation of the observed long-term trends in sea ice. As concluded by Polvani et al. [133], this appears to be the consequence of the relatively small fraction of variance explained by the seasonal coupling of the SAM and sea ice, which is surpassed by the larger fractions attributable to natural variations and the models’ internal variability. The effect of ozone depletion on changes in sea ice is, therefore, still not well understood.

Over much of the Antarctic continent, only relatively small seasonal changes in the short-wave albedo^20^ of the ice sheet occur and are primarily caused by deposition of snow and melting at the surface [146]. Local exceptions occur in the regions of exposed rock, which account for approximately 0.4% of the surface area of the continent. Here, varying coverage by ice, snow, and surface water can strongly influence albedo [147]. Changes in snowfall over Antarctica have been attributed to changes in atmospheric circulation resulting from the depletion of ozone [148], although patterns of relative change are heterogeneous [149].

### Associations between Arctic stratospheric ozone losses and the climate of the Northern Hemisphere

Years with a strong Arctic polar vortex and associated significant stratospheric ozone depletion have been linked to widespread climate anomalies across the Northern Hemisphere based on targeted model experiments with CCMs [150]. As an example, the exceptionally large ozone depletion that occurred in March–April 2020 (Sect. 3.3) not only led to record-breaking increases in Arctic solar UV radiation (Sect. 7.1.2) but also affected weather patterns in the Northern Hemisphere during spring. Specifically, it helped to keep the Arctic Oscillation (or AO^21^) in a record-high positive state through April [36], thus contributing to abnormally high temperatures across Asia and Europe [151]. Furthermore, loss of stratospheric ozone modified circulation patterns of winds around the Arctic, thereby affecting the stability of the upper troposphere in the Siberian sector of the Arctic. In turn, this led to more high-level clouds that enhanced downwelling long-wave (thermal) radiation [152]. The associated anomalous warming of the surface in April 2020 was further amplified by a reduction in albedo caused by melting of snow and sea ice. Monthly anomalies (relative to the 1981–2010 climatology) in air temperature of up to + 6 °C were observed over Siberia from January through May 2020 [153]. The temperature in the Siberian town of Verhojansk (68° N, 133° E) set a new record of 38 °C on 20 June 2020, which is the highest temperature ever documented near the Arctic Circle. Depletion of stratospheric ozone over the Arctic in March may cause reductions in the sea ice concentration and the sea ice thickness over the Arctic Ocean north of Siberia from spring to summer [154].

The unprecedented depletion of Arctic ozone in the spring of 2020 contrasts with the boreal winter of 2020/2021, when a major sudden stratospheric warming (SSW) occurred on 5 January 2021 [42, 43] and limited overall ozone loss (Sect. 3.3). During an SSW event, the westerly winds of the wintertime polar stratosphere decelerate and temperatures in the polar stratosphere rapidly increase [155]. The 2021 SSW event warmed the lower stratosphere, interrupted the catalytic cycles associated with ozone depletion [47], and also affected the polar atmospheric circulation from the upper stratosphere to the surface for six weeks after the event. During this period, surface temperatures were anomalously high over Greenland and the Canadian Arctic and anomalously low over Europe, northern Asia, and the United States, with a cold air outbreak first occurring over Eurasia in January and then over North America in the first two weeks of February [156]. SSWs generally increase the likelihood of such weather anomalies [157]; however, it is still unclear to what degree the cold weather events in early 2021 were linked to the SSW on 5 January 2021. There is some evidence that the cold outbreak in Siberia on 22–24 January 2021 was associated with the SSW [158]. However, simulations with a climate model did not find evidence that this SSW event caused or influenced the record-breaking cold in North America during February 2021.

Precipitation in Central China in April–May has been linked to Arctic stratospheric ozone changes in February–March by combining observations, reanalysis data, and a CCM [159]. Specifically, positive Arctic ozone anomalies enhance precipitation in central China and negative anomalies reduce precipitation. Another study, using the same CCM, demonstrated a negative relationship between Arctic ozone anomalies in March and surface temperature anomalies in central Russia and, a weaker positive relationship in southern Asia [160]. Furthermore, variations in precipitation occurring during April in the northwestern United States (mainly the states of Washington and Oregon) are strongly linked to changes in Arctic stratospheric ozone during March [161]. Specifically, higher-than-normal Arctic ozone concentrations in March lead to less precipitation in April and vice versa.

Despite these advances in the understanding, assessing linkages between Arctic ozone depletion and weather in the Northern Hemisphere remains difficult and is subject to large uncertainties. It is anticipated that future studies will refine the conclusions summarized above.

## Factors other than ozone affecting UV radiation

Solar UV-B radiation at the Earth’s surface is mostly controlled by the height of the Sun above the horizon (i.e., the solar elevation^22^); TCO; clouds; aerosols; the reflectivity of the surface, also called albedo; and altitude. Less important factors include: the vertical distribution of ozone in the atmosphere (i.e., the ozone profile) for fixed TCO; other trace gases such as sulfur dioxide (SO_2_) and nitrogen dioxide (NO_2_); seasonal changes in the Earth–Sun distance; changes in solar activity, which influence both stratospheric ozone concentrations and the UV-B irradiance at the top of the atmosphere; topography; and volcanic eruptions. Except for determinants related to the Sun and volcanic activity, all these factors are influenced by human activities—such as the release of GHGs and air pollutants—and are coupled with changes in the climate. For example, higher temperatures will lead to less sea ice in the Arctic, which will in turn reduce surface reflectivity and UV radiation at or above the surface. The effects of these factors have been described at length in previous assessments [9, 162, 163]. No studies published in the last four years provide new insights into the effect of clouds on UV radiation. We, therefore, focus in the following sections on new understandings into the roles of aerosols, albedo, solar activity, volcanic eruptions, and climate interactions on UV radiation.

### Aerosols

Natural and anthropogenic aerosols (solid and liquid particles suspended in the atmosphere) play a major role in controlling the intensity of UV radiation at the Earth’s surface. Although effects of aerosols have been discussed in numerous studies, the magnitude of these effects is still uncertain. The attenuation of surface UV radiation by aerosols depends on their amount, as measured by aerosol optical depth (AOD), and on their efficiency of absorption, as discussed at length in our last assessments [9, 162]. To quantify these effects further, Campanelli et al. [164] analyzed optical properties of aerosols and spectral irradiance in Rome, Italy, and correlated the variability of the UVI (adjusted for variations in TCO) with the AOD at 340 nm for two groups of either strongly or weakly absorbing aerosols. Absorption for the two classes was quantified with the single scattering albedo^23^ (SSA). For strongly absorbing aerosols (SSA < 0.9), an *increase* of the AOD by one unit resulted in a *decrease* of the UVI by 2.7 units (about 30%) for a solar zenith angle (SZA) of 30° and by 1.65 units (about 25%) for a SZA of 40°. For less absorbing aerosols (SSA > 0.9), the UVI decreased only by one unit (about 12%) per unit of AOD increase for both SZAs. The study illustrates the importance of the absorption properties of aerosols.

The paucity of measurements of the properties of aerosols (including the SSA) in the UV-B range [9] hampers our ability to accurately assess the effects of aerosols on a global scale as well as for urban regions with a diverse mix of aerosol types [5]. Global networks, such as the Aerosol Robotic Network (AERONET), which measure AOD and other aerosol properties, do not perform observations at UV-B wavelengths. While the technology for measuring AOD and SSA in the UV-B range exists and has been tested at a few sites [165–169], there are at present no reliable data to assess aerosol properties in this critical wavelength range on a global scale. However, the European Brewer Network (EUBREWNET) [170] has recently started to provide AOD in the wavelength range from 306 to 320 nm [167] and a preliminary analysis confirms the good quality of the data. It is anticipated that this network will expand globally.

**Fig. 5:**
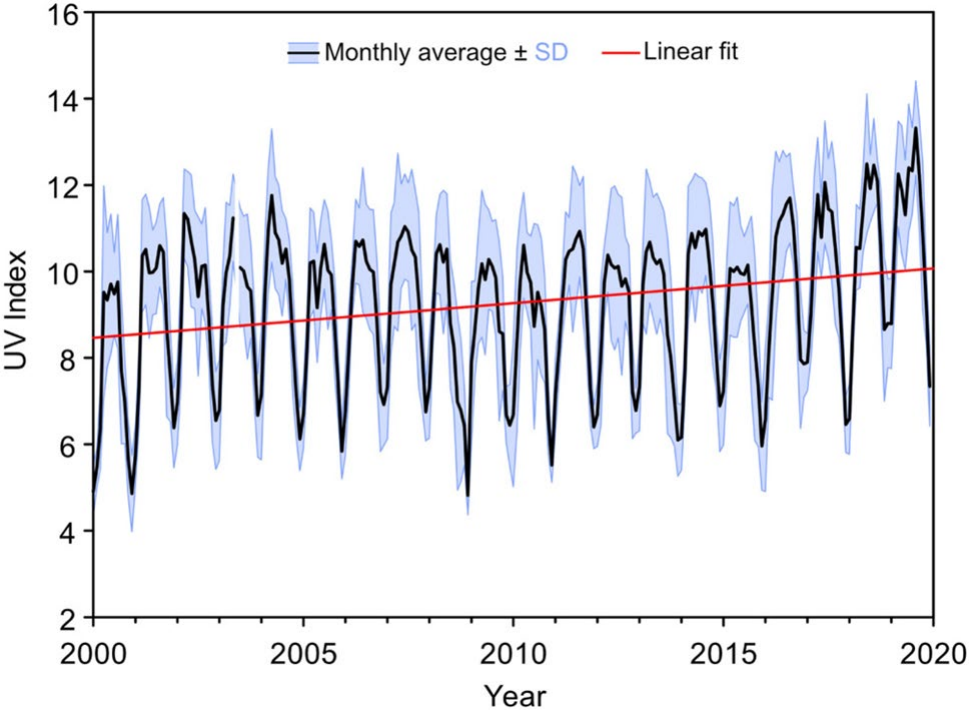
Monthly average noontime UVI in the Mexico City Metropolitan Area (black line) ± 1 standard deviation (blue shading), and linear fit (red line) to average data. Reprinted from Ipiña et al. [171] with permission from the American Chemical Society, Copyright © 2021

In areas with elevated levels of air pollution and small variability in TCO, the attenuation of solar UV radiation under cloudless skies is mainly controlled by aerosols. In such areas, abatement of air pollution can lead to increases in the intensity of UV radiation towards levels that would normally occur in unpolluted areas at similar latitudes and altitudes. An example of this is the observed increase of ~ 25% in UVI over Mexico City between 2000 and 2019, which was attributed to reductions in pollutants; in order of importance, aerosols, tropospheric ozone, NO_2_, and SO_2_ [171]. Because of high historical levels of air pollution in Mexico City, the UVI under cloud-free conditions was lower by ~ 40% in 2000 and ~ 25% in 2019 relative to values expected for an unpolluted clear atmosphere. Monthly averages of the daily maximum UVI from the 11 stations distributed across the Mexico City Metropolitan Area considered in this study show a clear upward trend of 0.9% per year between 2000 and 2019, and an overall increase in monthly maximum UVI of 1.5 over the two decades (Fig. 5). Since 2016, the rate of increase is greater, possibly reflecting more aggressive measures in reducing air pollutants. Human health benefits resulting from the decrease in air pollution [5, 172] outweigh risks—such as the potential increase in skin cancer incidence—stemming from the gradual return of UV radiation intensities to more natural levels prevailing at unpolluted areas^24^.

The effects of air quality measures implemented in Mexico City may help to project changes in UV radiation for regions that are currently still affected by heavy smog, such as South and East Asia [9, 173]. Finally, the study for Mexico City also confirmed earlier findings [e.g., 174] that the UVI at the surface of heavily polluted areas cannot be reliably estimated from satellite observations, emphasizing the importance of ground-based measurements. A similar finding was reported by Roshan et al. [175] for the city of Doha, Qatar, when extreme dust storms resulted in a measured UVI of 6–7 compared to a UVI of 10–11 estimated by the OMI satellite on the same days.

The effect of increased aerosols and tropospheric ozone on surface UV radiation during the biomass burning^25^ season in Pretoria, South Africa, was investigated by du Preez et al. [176]. The simulations included different scenarios with and without increased levels of aerosols and tropospheric ozone from biomass burning. For cloudless days during the height of the biomass burning period in September, aerosols and tropospheric ozone reduced the noontime UVI by 13% and 1%, respectively, demonstrating that changes in the UVI were dominated by the effects from aerosols.

Smog from the Black Summer wildfires in Australia (Sect. 5.1.2) led to extreme air pollution and low visibility. However, even during days with a visibility of less than 5 km, the intensity of UV radiation may have still been harmful to human health. For example, on 10 December 2019, the visibility near Sydney, Australia, dropped to about 1 km around noon. Despite this low visibility, the cumulative erythemal UV dose measured at this location over a one-hour period at noon was still more than 4 SED^26^ or about 46% of the one-hour dose measured on the cloud and haze-free day of 27 November 2019. During the eight hours between early morning and late afternoon the dose on 10 December 2019 was 17 SED. The corresponding dose of 27 November was 48 SED [180]. These UV doses far exceed the maximum daily UV dose recommended by ICNIRP for outdoor workers [181]. While most people stayed indoors during the fires because the air pollution was so extreme, emergency workers, who had to be outside despite adverse conditions, may have been exposed to UV radiation levels harmful to human health, potentially without being aware of it and without applying appropriate sun protection measures.

Despite increases of aerosols in specific regions (e.g., from bushfires, burning of biomass or dust storms), over most populated areas of the globe, there is a general decrease in aerosols. Trends of aerosol optical and chemical properties on global and regional scales have been reported from observations with several ground-based networks [182]. Most of the properties related to loading of aerosols exhibit negative trends in the period 2000–2014 in regions covered by observations, both at the surface and in the total atmospheric column. Significant decreases in AOD were found in areas with intense anthropogenic activity (Europe, North America, South America, North Africa and Asia), ranging from − 1.2% per year to − 3.1% per year. These data were used to validate various aerosol models (six AeroCom^27^ phase III models, four CMIP6 models and the CAMS^28^ reanalysis dataset) showing good agreement in the AOD trends. When these models were used to estimate the global AOD trend by filling the gaps in regions not covered by observations, a global increase in AOD of about 0.2% per year between 2000 and 2014 was found, primarily caused by an increase in the loads of organic aerosols, sulfate, and black carbon. These findings highlight differences between regional and global effects of aerosols on UV radiation, which must be considered, especially when projecting into the future.

In a modeling study [183] exploring China’s future anthropogenic emission pathways, it was projected that emissions of major air pollutants (i.e., SO_2_, NOx, PM_2.5_ aerosols, and non-methane volatile organic compounds) in China will be lower by 34–66% in 2030 and by 58–87% in 2050 compared to 2015. These estimates were derived by considering a combination of strong low-carbon and air pollution control policies. A second study [184] investigated the evolution of different types of aerosols over the Euro-Mediterranean region between 1971–2000 and 2021–2050 according to three different scenarios representing a wide range of possible future pathways. The study showed a decrease in AOD of between 30 and 40% over Europe, mainly from decreasing emissions of sulfur dioxide. However, these reductions are partly (~ 30%) compensated by increases in the optical depth from nitrate and ammonium particles.

Attenuation of UV radiation by aerosols can sometimes also mask the effect of “ozone mini-holes” (defined as a synoptic-scale^29^ region with strongly decreased TCO resulting from dynamical processes [185]) that would otherwise lead to increases in UV radiation. One example is an event that occurred in Athens, Greece, during 8 days in May 2020 [186]. On 15 May 2020, TCO was 43 DU (or more than 2 standard deviations) below the climatological mean, which would have normally led to an increase in the UVI by ~ 29%. However, the AOD on this day was 0.31 (47%) higher than the climatological mean due to the intrusion of Saharan dust, and measured UVIs agreed to within ~ 2% with the climatological mean. Hence the opposing effects of low TCO and high AOD nearly canceled each other. This study highlights the important role of aerosols in modifying the effects of changes in TCO on surface UV-B radiation. There is some evidence that the weather pattern that led to the transport of dust from Africa towards Athens was also responsible for the occurrence of the ozone mini-hole and the low TCO over Athens that ensued.

### Surface reflectivity

Changes in the reflectivity of the Earth’s surface (both land and ocean) can change the downwelling UV radiation because radiation that is reflected upward by the surface may subsequently be scattered downward by air molecules, aerosols, and cloud droplets. Topography can modify the reflectivity resulting in complex effects on UV radiation, as for example in narrow valleys with snow covered slopes. The largest effect of surface reflectivity occurs in areas with variable snow and ice cover because of the large difference in the albedo of bare and snow/ice-covered ground. This variability is often linked to climate change. For example, because of the warming of the Arctic, the start date of the spring snow melt at Ny-Ålesund (79° N), Svalbard, has advanced by three days per decade over the last 40 years [187], so now begins about two weeks earlier than in the early 1980s.

**Fig. 6:**
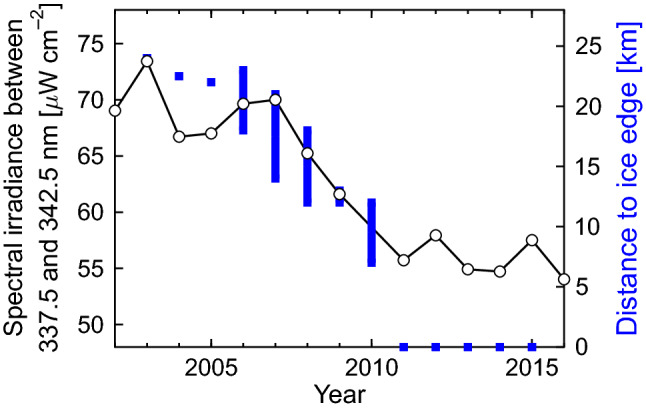
Comparison of monthly mean spectral irradiance between 337.5 and 342.5 nm for March (left axis) at Arrival Heights, Antarctica, and approximate distance of the outer edge of the land-fast ice from Arrival Heights (right axis) during March. Distance data are based on Fig. 3 of Kim et al. [189]. The vertical extension of the blue bars indicates the variability of the distance within the month of March. A distance of zero km from the ice edge means that McMurdo Sound, 1 km west of Arrival Heights, was free of ice. In years when the ice edge was far from the station and the ocean surrounding the station was covered by sea ice, the albedo was greatly enhanced and UV radiation in these years tended to be higher compared to years when McMurdo Sound adjacent to the station was free of ice. Adapted from Bernhard and Stierle [190]

Figure 6 illustrates the effect of surface albedo on UV radiation by comparing UV irradiance in the 337.5–342.5 nm range measured at Arrival Heights (78° S), Antarctica, with the extent of land-fast ice—defined as sea ice fixed in place by attachment to land, glaciers, grounded icebergs, or ice shelves—covering McMurdo Sound 1 km west of Arrival Heights. In 2000, a mega-iceberg calved from the Ross Ice Shelf, became temporarily trapped, and persisted in the entrance to McMurdo Sound for five years [188]. The tabular iceberg interrupted the normal movement of sea ice, resulting in McMurdo Sound remaining covered by ice with high albedo until April in some years [189]. As a consequence, UV irradiance was elevated in March between 2001 and 2007 when the ice edge was more than 13 km away from McMurdo, while less UV radiation was observed between 2011 and 2015 when McMurdo Sound was free from land-fast ice [190]. Since similar data are not available before 2000 and after 2016, sea ice cannot be correlated with UV radiation over a longer time period at this location. However, Kim et al. [189] reported that the dates of the retreat of land-fast ice in McMurdo Sound have not changed over the last 37 years except for years affected by mega-icebergs.

### Solar activity

Variability in the solar activity can indirectly affect UV radiation at the Earth’s surface through changes induced in atmospheric ozone, particularly in the stratosphere. These changes in ozone are caused by two different mechanisms, which are both related to the 11-year variability of solar activity. One mechanism is mediated through photochemical processes in the upper atmosphere that are modified by changes in solar UV-C (100–280 nm) radiation. The other process is driven by changes in the rate of energetic particle^30^ precipitation (EPP), which mainly affect ozone over the polar regions [191, 192].

The increase in emissions of solar UV-C radiation between the minimum and maximum of the solar cycle leads to increases in ozone concentrations in the upper stratosphere (altitude of 30–60 km) and decreases in the lower stratosphere (15–30 km), mainly at lower latitudes [193]. Using a CCM, Xiao et al. [194] estimated that for a 5% (10%) increase in solar output in the spectral range of 200–370 nm, the globally averaged ozone increases by up to 4.5% (9.0%) in the upper stratosphere, and decreases by up to 1.5% (3.3%) in the lower stratosphere. It was further noticed that the response of ozone to the variability of UV-C radiation during a solar cycle is non-linear, confirming earlier results [195].

Our previous assessment [9] discussed the effects of reduced solar activity in the future (e.g., from a Grand Solar Minimum^31^) on UV-B radiation received at the Earth’s surface. Based on the work by Arsenovic et al. [196], we concluded that UV-B radiation at the top of the atmosphere would decrease slightly due to weaker emission from the Sun; however, the reduced solar activity would also lead to decreases of ozone production in the stratosphere, resulting in an overall increase of UV-B radiation at the surface. This conclusion is still valid.

Solar activity has recently shown a declining tendency, suggesting that the Sun has entered into a modern Grand Solar Minimum period, from about 2020 to 2053, which would lead to a significant reduction of the solar magnetic field and magnetic activity by about 70%, similar to the Maunder minimum that occurred in the period 1645–1710 [197]. The influence of such reductions in total solar irradiance (TSI^32^) on surface temperatures was investigated using a climate model run under the RCP 8.5 scenario, which predicted a decrease in the global average temperature for the second half of the twenty-first century of 0.13 °C due to atmospheric effects of the upcoming Grand Solar Minimum [198]. Simulations by Arsenovic et al. [196], which were based on the RCP 4.5 GHG scenario, estimated that a stronger solar minimum with reduction in TSI of 0.48% would only compensate for about 15% of GHG-induced warming by 2100. Hence, the estimated decreases in temperature by 2100 due to reduced solar activity are small compared to the projected increases due to GHG emissions. Therefore, the reduction of solar irradiance during a possible Grand Solar Minimum would only partly offset the anthropogenic change in climate caused by continuing GHG emissions.

The upcoming maximum of Solar Cycle 25 is expected to be weaker than the current Cycle 24, which was the weakest in at least the past 100 years [199, 200]; however, the uncertainty of this prediction is large. Model results [199] estimated a deep extended solar activity minimum for 2019–2021, and a weak solar activity maximum in 2024–2025. This modeling study is based on analysis of magnetograms that contain information on the evolution of magnetic fields on the solar surface, allowing forecasting of the solar activity in the future. The reduced activity in the period of the solar maximum will lead to less photochemical production of stratospheric ozone at low latitudes, but also to reduced polar ozone destruction due to fewer energetic particles.

Although none of the studies discussed above addressed effects on surface UV-B radiation, the upcoming weaker solar activity period would lead to decreases in stratospheric ozone and consequently to increases in UV-B radiation at the surface, despite the reduced solar irradiance entering the Earth’s atmosphere. This effect has not been considered in the projections of UV radiation described in Sect. 8.

### Volcanic eruptions

Throughout the Earth’s history, major volcanic eruptions or impacts of meteors have perturbed the climate, affected the stratosphere, and caused regional and global environmental disasters. Global effects are mainly caused by the reflection of incoming solar radiation by the aerosol layer that forms in the stratosphere after a large volcanic eruption, but also by the destruction of stratospheric ozone involving heterogeneous chemical reactions on the surfaces of volcanic aerosols in the presence of halogens [201–203]. Volcanic aerosols are dispersed zonally to other latitudes and can persist for several years, resulting in cooling of the troposphere. Such eruptions can either reduce solar UV-B irradiance at the Earth’s surface through scattering of radiation back to space or increase it through reduced absorption by the depleted ozone layer. The magnitude of these effects depends on the strength of the eruption and on the amounts of aerosols and halogenated compounds involved. Large tropical volcanos in the last ~ 200 years, e.g., Mt. Pinatubo in 1991 and Mt. Tambora in 1815, have caused globally averaged cooling of 0.3 and 0.7 °C at the Earth’s surface, respectively [204]. Conversely, stratospheric aerosols from Mt. Pinatubo warmed the lower tropical stratosphere by up to 4 °C in the 2–3 years following the eruption [205].

Recent studies used chemistry–climate models to investigate the effects of different amounts of SO_2_ and halogens injected into the tropical stratosphere by volcanic eruptions [204, 206, 207]. Brenna et al. [206] assumed an explosive eruption at 14° N, rich in sulfur, chlorine, and bromine compounds, occurring during pre-industrial times. The assumed amount of SO_2_ injected represents the average of 28 historical volcanic eruptions in the Central American Volcanic Arc (CAVA; extending parallel to the Pacific coastline from Mexico to Panama), comparable in magnitude with the Mt. Pinatubo eruption. However, the amount of bromine and chlorine deposited in the stratosphere was assumed to be much larger than the amount estimated for Mt. Pinatubo. (The Mt. Pinatubo eruption was unusual because it occurred at a time when the Philippines were also inundated by a typhoon. Water droplets from the storm likely adsorbed halogen compounds in the plume and prevented them from reaching the stratosphere [208, 209].) The ozone depletion calculated by Brenna et al. [206] led to increases in the clear-sky summertime UVI of more than 50% in the NH during the first two years after the eruption. Maximum increases in the UVI were modeled to exceed 7 units in the NH tropics and subtropics, and peak at 4 units in the NH mid-latitudes. Much of the mid-latitudes would have experienced a UVI above 15, which is similar to present-day peak values in the tropics [210]. This simulation was based on the injection of large amounts of halogens, which are thought to be representative for volcanic eruptions in the CAVA. Simulated increases in UV radiation are therefore much larger than those observed after the eruption of Mt. Pinatubo.

Another modeling study [204] investigated the effect on the atmosphere of the eruption of a super volcano like Toba, which erupted 74,000 years ago. It has been estimated that Toba injected 100 times more SO_2_ into the stratosphere than Mt. Pinatubo. According to this study^33^, such an event could lead to the collapse of the ozone layer in the tropics with ~ 50% reduction in TCO, which would increase the daily maximum UVI by more than a factor of two. Even with one fifth of the injected SO_2_ amount, ozone depletion in the tropics would be similar to that currently occurring in Antarctica and would last for nearly a year.

These studies show that massive but rare volcanic eruptions can lead to severe depletion of stratospheric ozone, changes in atmospheric circulation and temperature patterns, and large increases in UV-B radiation at the Earth’s surface. These increases can by far exceed those associated with ozone depletion from ODSs in the 1980s and 1990s as well as the expected rise in UV-B radiation to more natural levels over urban regions that may occur when measures to reduce air pollution are implemented (Sect. 6.1).

### Climate change

In the absence of changes in the TCO, climate-change-induced trends in the properties of clouds, atmospheric aerosols and surface albedo have the potential to strongly influence the long-term behavior of UV radiation at the Earth’s surface.

The optical properties of clouds, aerosols, and surface albedo, and the interactions between these components, are active areas of research because of their importance in the radiative balance at the surface. Global warming is expected to influence cloudiness because of the atmosphere’s ability to hold more water as temperatures increase [211]. However, patterns of change in cloud cover, height, and optical depth are difficult to assess because of the inherent internal variability in regional climate forcing combined with the short length of available climate data records. The physical understanding of cloud processes continues to advance. For example, the better understanding of the microphysics of supercooled liquid water has reduced the bias in the modeled short-wave cloud radiative effect over the Southern Ocean [212]. Climate models also continue to improve in their representation of aerosols, which cool the lower troposphere and counter some of the warming resulting from GHGs [63]. Reductions in air pollution have generally occurred in Europe and North America as the result of regulations; however, economic growth has caused large regional increases in aerosol emissions in Asia and Africa [213]. Interactions between aerosols and clouds remain the largest uncertainty in climate projections. Changing patterns of coverage of the surface with snow, ice, and vegetation under global warming are also relevant to surface UV irradiance, with observed darkening of the Arctic surface over 2000–2019 attributed to summertime loss of sea ice, while mixed trends in albedo have occurred over this period in Antarctica [214] (see also Sect. 6.2).

The complexity in accurately accounting for all relevant processes, particularly on small scales where observations are influenced by local effects (e.g., UV enhancement under broken clouds), limits the ability to attribute trends in UV irradiance to specific climate change effects. However, several recent studies have quantified local-scale influences, with examples provided below.

The occurrence of cloud-free conditions is very important for total UV exposure. Atmospheric blocking systems, which are large-scale patterns of stationary atmospheric pressure fields that “block” or redirect migratory cyclones or anti-cyclones, can lead to prolonged periods of clear skies at mid and high latitudes. In a blocking event, a high-pressure weather system can persist for days or even weeks over some geographical regions, inhibiting cloud formation and causing moisture in the westerly zonal flow to be deflected around it. Hence, clouds are often more persistent than usual outside regions with high pressure resulting in lower UV irradiance at the Earth’s surface. A recent example where surface UV radiation was exceptionally affected by atmospheric blocking occurred during May–July 2018 in Norway and Finland [215]. The monthly mean noontime UVI was 20–40% above the long-term mean as a direct result of decreased cloud cover. For example at Sodankylä (67° N), the mean temperature in July 2018 was 5.6 °C above the 1981–2010 average for the same month and the duration of sunshine in 2018 was 405 h, exceeding the 1981–2010 average of 245 h by 65%. This particular event was associated with a record heat-wave in central and northern Europe [216]. Recent studies examining trends and variability in atmospheric blocking at high latitudes have found mixed patterns of change, with regional shifts in trends in the Antarctic Peninsula region over the satellite era [217], and no significant trends over Greenland [218]. For high-emissions SSP scenarios, a clear decrease in future blocking over Greenland and the north Pacific was found, but seasonal and regional projections are generally unclear [219].

It has been known for decades that changes in tropopause height are inversely linked to changes in TCO [220, 221]. If the tropopause is shifted up, some lower stratospheric ozone is horizontally transported to surrounding regions with lower tropopause height. The result is a decrease of TCO in areas where the tropopause is elevated [221]. Furthermore, mid-latitude regions with elevated tropopause may also be influenced by the advection of stratospheric ozone-poor air masses from lower latitudes (ozone mini-holes) [185, 222].

In a new study, Fountoulakis et al. [223] quantified the effect of changes in the geopotential height (GPH) at 250 hPa (a quantity similar to tropopause height) on TCO and spectral irradiances at 307.5 and 324 nm at three locations across Italy: Aosta (46° N), Rome (42° N), and Lampedusa (36° N). Statistically significant anti-correlations were found between GPH and monthly anomalies in TCO for all locations and months. Conversely, positive correlations between GPH and monthly anomalies in spectral irradiance at 307.5 nm were detected for most months. The study makes a strong case that increases in GPH or tropopause height that are expected from the warming of the troposphere due to climate change [224, 225] would reduce TCO and subsequently lead to increases in UV-B radiation.

Additional effects of climate change on TCO and the vertical distribution of ozone in the atmosphere—such as the expected strengthening of the Brewer-Dobson circulation, unexpected declines in lower stratospheric ozone in the extratropics [226], and the dependence of TCO on GHG scenarios (Sect. 3.5)—are discussed in great detail in SAP’s latest report [11] and are therefore not addressed here.

## Variability in UV radiation and trends from observations

This section assesses observed variations in UV radiation on various time scales as well as long-term trends in the UVI observed by ground-based and space-borne instruments over several decades.

### Variations in UV radiation with time and altitude

Year-to-year and seasonal variability in UV radiation is mainly controlled by variations in the TCO, cloud cover, and aerosols. For example, TCO at mid-latitudes is higher in the spring and lower in the autumn. As a result, the UVI near the autumn equinox can exceed that at the spring equinox by nearly a factor of two for matching SZAs [227]. The effect from ozone is most pronounced at high latitudes of the Southern Hemisphere during spring but variability in stratospheric ozone in the Arctic has also led to larger variability in UV radiation at northern high latitudes in recent years during the late winter and early spring season. Both regions are discussed in the following sections.

#### Temporal variations of UV radiation in Antarctica

We reported in our previous assessment [9] that the variability of UV-B radiation in Antarctica observed between 2014 and 2017 was very large, with near record-high UVIs observed at the South Pole in spring 2015, and well below average values in spring 2016. Variability during the period discussed in this report (2018–2021) was equally large, despite evidence that stratospheric ozone concentrations over Antarctica are now recovering (Sect. 3.2).

**Fig. 7:**
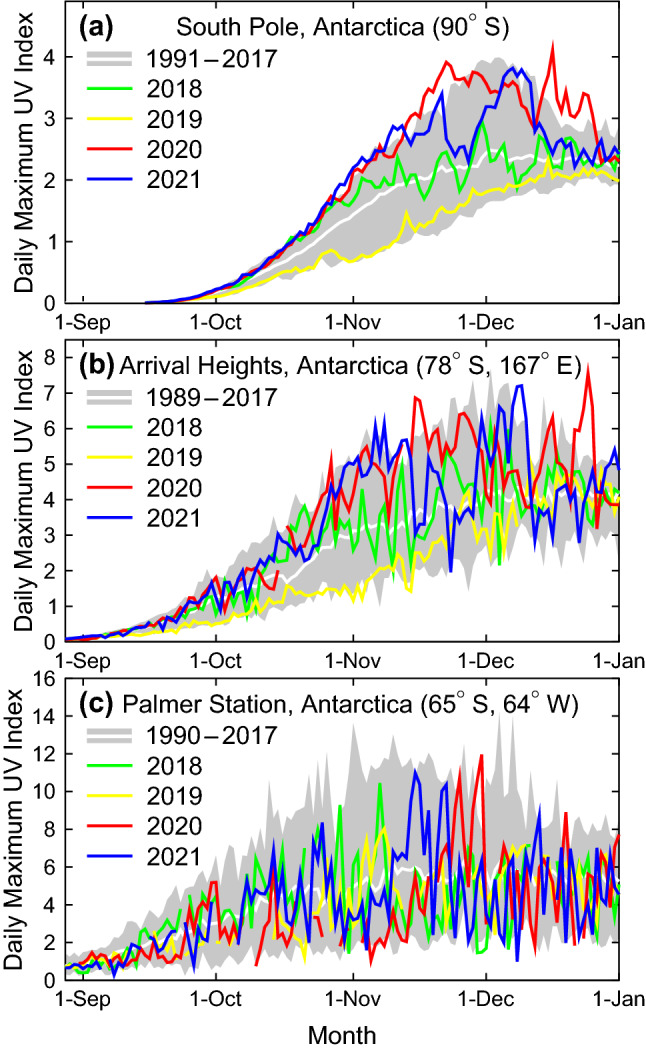
Daily maximum UVI measured at **a **the South Pole, **b** Arrival Heights, and **c **Palmer Station in 2018 (green), 2019 (yellow), 2020 (red), and 2021 (blue) compared with the average (white line) and the range (gray shading) of daily maximum observations of the years indicated in the legends. The UVI was calculated from spectra measured by SUV-100 spectroradiometers. Up to 2009, the instruments were part of the NSF UV Monitoring Network [228] and they are now a node in the NOAA Antarctic UV Monitoring Network (https://gml.noaa.gov/grad/antuv/). Consistent data processing methods were applied for all years [190, 229]

Figure 7 shows the daily maximum UVI observed at three Antarctic stations (South Pole (90° S), Arrival Heights (78° S), and Palmer (65° S)) for September–December, the months most affected by the ozone hole. Observations in 2018, 2019, 2020, and 2021 were compared with the average and range of measurements between ~ 1990 and 2017. UVIs in October 2018 were well above the long-term mean and approached historical maxima at the South Pole but remained within the range of typical variability at the other two sites. Conversely, unusually low UVIs were observed at the South Pole and Arrival Heights in spring 2019 due to a record-high TCO during this period. Between October and mid-November 2019, the UVI at the South Pole was at the minimum of the historical (1991–2017) range and remained close to this minimum between mid-November 2019 and January 2020. At Arrival Heights, the UVI in 2019 was close to the minimum between September and mid-November, and stayed below the long-term mean until mid-December, except for two short periods.

In contrast to 2019, near record-high UVI maxima were observed in spring 2020 and 2021 because of large and persistent Antarctic ozone holes in these years (Sect. 3.2). In both years, the UVI at the South Pole tracked or exceeded the historical range between September and mid-November and set new records in mid-November and mid-December 2020. On 21 November 2020, the maximum UVI measured on this day exceeded the average of the daily maxima for 21 November, calculated from measurements of the years 1991–2017, by 83%. At Arrival Heights, the UVI reached a new all-time site record of 7.8 on 23 December 2020, exceeding the previous record for this day by nearly 50%. Measurements at Palmer Station were highly variable, as is typical for this site, but new records were also set at this site in the second half of November 2020 when the center of the ozone hole was above the station. High UV radiation at this time, which coincides with the start of the growing season for plants and the peak breeding season for most animals, is a concern [3].

The record-high UVIs in 2020 were not only confined to the three stations shown in Fig. 7 but also observed at other Antarctic research stations. At the Australian Antarctic bases Casey (66° S), Mawson (68° S), and Davis (69° S), UVIs measured with broadband radiometers between October and December 2020 were generally well above the 2007–2019 climatological mean, with new record-high values set on several days in November and December [31]. The number of days when TCO dropped below 220 DU and led to spikes in UVI was the highest ever observed at the three sites. The daily maximum UVI at Marambio, a station located near the Antarctic Peninsula at 64° S, exceeded 12 on several days in late November and early December 2020 [20]. Similarly, extreme UVI values were measured at King George Island (62° S), near the northern tip of the Antarctic Peninsula [34]. The UVI exceeded 11 on four days between 24 November and 4 December 2020 and peaked at 14.3 on 2 December. This value ties, within the measurement uncertainty, with the highest value of 14.2 (recorded on 4 December 1998) ever measured at Palmer Station ([230] and Sect. 7.3). On 3 December 2020, the erythemal daily dose at King George Island was 8.1 kJ/m^2^, which is among the highest on Earth and only comparable to those recorded at high-altitude sites such as the Atacama Desert, Chile [231], or at Mauna Loa, Hawaii, where the highest dose ever observed was 9.5 kJ/m^2^ [232]. These extreme levels of UV radiation were a result of solar elevations close to their annual maximum; close to 24 h of daylight at King George Island; broken clouds, which can enhance radiation levels at the surface beyond the clear-sky level when the solar disk is free of clouds and additional radiation is scattered by clouds to the observer; and low TCO. For example, on 1 December 2020 the TCO over King George Island was 180 DU, which is the lowest value ever recorded for December at this site [34]. UVI data for 2021 from stations other than those shown in Fig. 7 are not yet available.

The findings of the studies discussed above show that variability of springtime UV-B radiation in Antarctica is large despite ongoing reduction of ODSs and signs of ozone recovery. This surprisingly high variability is mainly driven by changes in meteorological conditions and in particular the persistently low temperature of the lower stratosphere.

When the Antarctic polar vortex breaks up at the end of the austral spring, ozone-depleted air masses disperse to lower latitudes, which may lead to large increases in UV radiation over populated areas in the Southern Hemisphere [233]. However, a recent study found that the breakup of the polar vortex had only a small effect on UV radiation at Cape Town, South Africa (34° S). Elevated levels of UV radiation at this location were more frequently associated with low-ozone air masses of tropical origin [234].

#### Temporal variations of UV radiation in the Arctic

As discussed in Sect. 3.3, an exceptionally large episode of stratospheric ozone depletion was observed in late winter and early spring (February–April) of 2020 in the Arctic [35, 36, 39, 41].

**Fig. 8:**
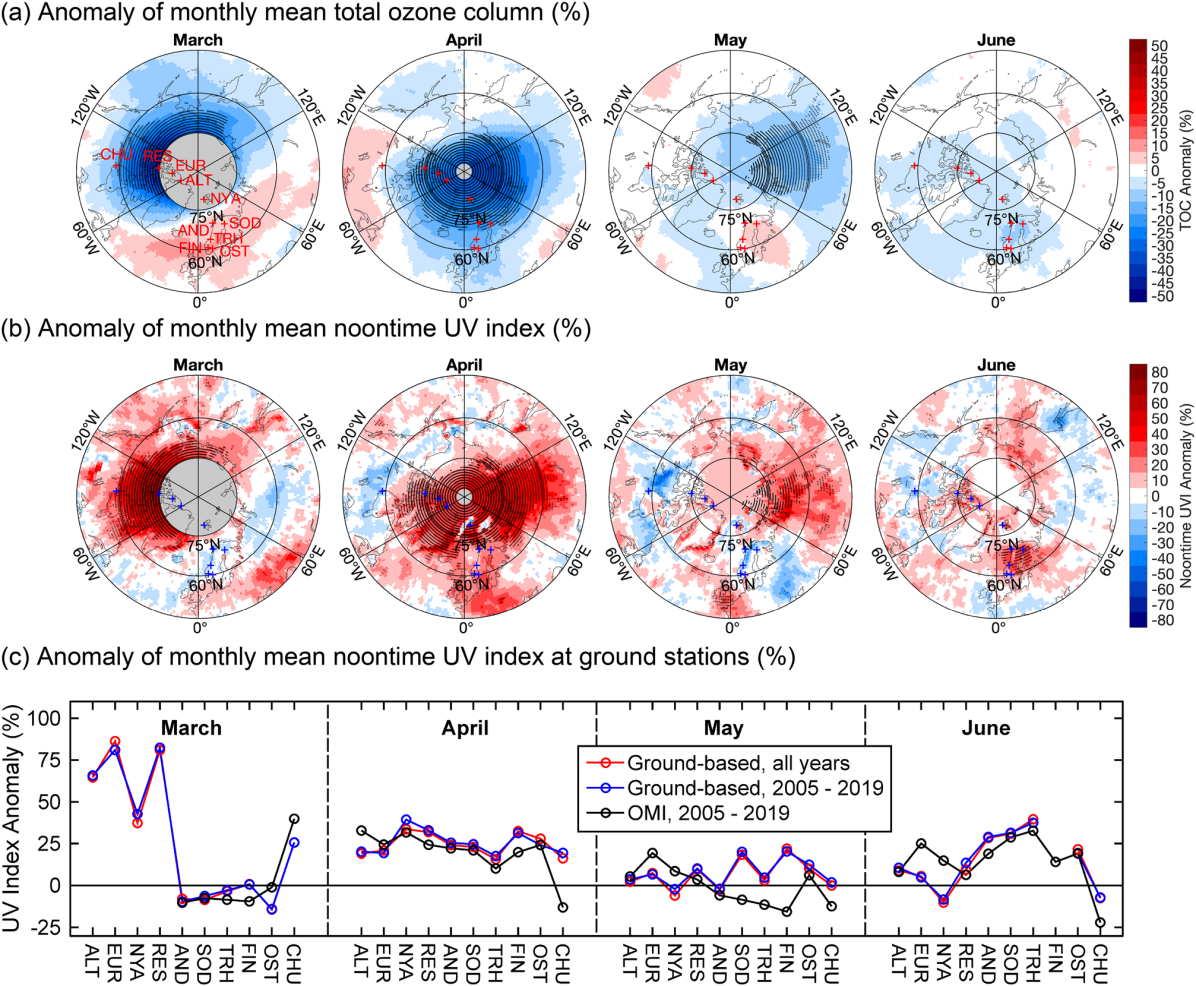
Monthly mean anomaly maps (in %) of **a **TCO and **b** noontime UVI for March, April, May, and June 2020 relative to 2005–2019 means. Stippling indicates pixels where anomalies exceed three standard deviations (3*σ*). Gray-shaded areas centered at the North Pole in the maps for March and April indicate latitudes with no OMI data because of polar darkness. Locations of ground stations are indicated by crosses in every map, with labels added to the first panel. Maps are based on the OMTO3 Level 3 TCO product [237]. **c** Percentage anomalies in monthly means of the noontime UVI for 2020 derived from measurements at 10 ground stations (North to South along the x-axis) relative to all years with available data (red) and 2005–2019 (blue). The black datasets indicate anomalies for the same stations derived from OMI measurements relative to 2005–2019. Site acronyms are ALT: Alert (83° N); EUR: Eureka (80° N); NYA: Ny-Ålesund (79° N); RES: Resolute (75° N); AND: Andøya (69° N); SOD: Sodankylä (67° N); TRH: Trondheim (63° N); FIN: Finse (61° N); OST: Østerås (60° N); and CHU: Churchill (59° N). Figure adapted from Bernhard et al. [235]

Figure 8a shows deviations of monthly average TCO from past (2005–2019) averages north of 45° N for March, April, May, and June, and their effects on UVI. In March 2020, relative TCO anomalies of up to − 40% and exceeding 3 standard deviations (*σ*) were measured over northern Canada and the adjacent Arctic Ocean. In April, relative TCO anomalies of up to − 35% and exceeding 3σ were observed for virtually all areas north of 60° N. During the breakup of the polar vortex in May [35], areas with abnormally low (> 3*σ*) TCO still persisted over Siberia.

The low TCO led to record-breaking anomalies in solar UV-B radiation over the Arctic measured by ground-based instruments at ten Arctic and subarctic locations and observed by the Ozone Monitoring Instrument (OMI) on NASA’s Aura satellite [235, 236]. *Relative* UV-B radiation anomalies were particularly large between early March and mid-April 2020. However, *absolute* anomalies for this period remained small (e.g., below 0.6 UVI units) because solar elevations for March and April are still low in the Arctic. In the following, we only discuss relative anomalies.

In March 2020, the monthly average UVI over the Canadian Arctic and the adjacent Arctic Ocean was between 30 and 70% higher than the historical (2005–2019) averages, often exceeding the climatological average by 3σ. By April 2020, they were positive over a vast area, including northern Canada, Greenland, northern Europe, and Siberia. The maximum anomaly was 78% and anomalies exceeded 3σ almost everywhere north of 70° N. In May 2020, UVI anomalies of up to 60% and exceeding 3σ were measured over Siberia. The UVIs in June were elevated by up to 30% over parts of Norway, Sweden, and Finland, resulting from a combination of negative TCO anomalies and unusually fair weather with several cloudless days [236]. Ground-based measurements generally confirm UVI anomalies derived from satellite data (Fig. 8c). However, notable differences between the ground-based and satellite data sets exist for Sodankylä (Finland), and Trondheim and Finse (Norway) in May. These discrepancies are likely caused by a mismatch between the albedo climatology used in the satellite retrieval and the actual albedo. Albedo in May is affected by the timing of snow melting, which was unusually late at Sodankylä and Finse in 2020.

In contrast to 2020, Arctic UVI anomalies in 2019 and 2021 remained within 2σ of the climatological mean, with few exceptions [46, 47]. One exception is the large UVI anomalies of up to 65% in the period 15–30 April 2019 in Norway, Sweden, and Finland, when a persistent high-pressure system with clear skies was centered over the Nordic countries. As the TCO in the Arctic is projected to have large year-to-year variability for the remainder of the twenty-first century, large variations in UV radiation are likely to occur over the next decades.

#### Dependence of UV radiation on altitude

Measurements from satellites suggest that the highest UVI values observed during the year at the Earth’s surface range from less than 3 at the poles to about 25 at high altitudes within the tropics of the Southern Hemisphere, such as the Altiplano Region of Peru and Bolivia [210]. The average altitude of this region is 3750 m and the highest peak (Illimani) is at 6438 m above sea level (asl). Ground-based measurements of UV radiation in this area are sparse despite their importance for human health and ecosystems. Recent measurements at Quito, Ecuador (2850 m asl), established a maximum UVI of 21 at this location [238]. This value is consistent with the highest value of 21.2 measured at Mauna Loa (3397 m asl) [232] and supports the maximum value of ~ 25 for the highest UVI that may occur on Earth considering that Quito is at a considerably lower elevation than the highest peaks of the Andes. The extreme UVI values at high-altitude locations close to the equator may have significant health effects for people moving to these regions for work or recreation without taking appropriate precautions to protect themselves from UV radiation [239].

### Observed long-term changes in UV radiation

In the last four years, new trends in UV radiation derived from ground-based measurements have been published for several regions [190, 223, 238, 240–245]. These studies confirm that changes in UV radiation during the last 25 years have generally been small—typically less than 4% per decade, increasing at some sites and decreasing at others, with few exceptions—consistent with the multi-site study by McKenzie et al. [56] discussed in Sec 4.1. Results from these studies are assessed in more detail below. While only studies that appeared to be of high quality according to our assessment were included, the measurement uncertainty of the various datasets varies and the reader is referred to the original publications for details.

Trends in solar spectral irradiance at 307.5 nm, which is a reasonable proxy for trends in erythemally weighted UV radiation, were calculated at several stations in Europe, Canada, and Japan over a 25-year (1992–2016) period [240]. Long-term changes at this wavelength vary by location and are mostly driven by changes in aerosols and TCO. However, at high northern latitudes, changes in the surface reflectivity are also an important factor. Over Japan, the spectral irradiance at 307.5 nm has increased significantly by about 3% per decade over this 25-year period and this increase is attributed to a decrease in absorbing aerosols. The only European station with a significant trend was Thessaloniki, Greece, where spectral irradiance at 307.5 nm rose by 3.5% per decade with an increasing rate of change during the last decade, possibly because of decreasing absorption by aerosols.

Updated estimates of trends in UV-B irradiance at four European stations (Reading (51° N), Uccle (51° N), Sodankylä (67° N), and Thessaloniki (41° N)) have been reported for the period 1996–2017, i.e., starting after the global peak of ODSs [245]. The study concluded that the variability of UV-B radiation at these European sites was mainly governed by variations in clouds, aerosols, and surface reflectivity, while changes in TCO were less important. Statistically significant (95% confidence level (CL)) positive trends in noontime spectral irradiance at 307.5 nm were found for Thessaloniki (8% per decade) and Uccle (5% per decade), while, for Reading, the trend was negative (− 7% per decade). These trends were again attributed to the effects of aerosols and clouds. No statistically significant trend was found at Sodankylä; however, the decreasing tendency of − 5% per decade at this site was found to be consistent with changes in surface reflectivity due to declining snow cover in late winter and spring. In a follow-on study [223], a similar trend analysis was performed for Rome (42° N), Italy. A statistically significant negative trend in TCO of –1% per decade was found, but there was no corresponding significant increase in spectral irradiance at 307.5 nm over the period 1996–2020. However for certain months, positive trends in UV irradiance were observed, which were predominantly caused by changes in clouds and/or aerosols.

Several other studies reported estimates of trends for erythemal irradiance (or erythemal doses) at northern European sites. No statistically significant trends in erythemal UV radiation were observed at Moscow, Russia (56° N), over the period from 1999 to 2015 [243]; at Chilton, England (52° N), between 1991 and 2015 [246]; and at Tõravere, Estonia (58° N), between 2004 and 2016 [244]. At the last site, there were also no trends in the main factors influencing UV radiation, namely TCO; aerosol optical depth; and global short-wave radiation, which is a proxy for the effect of clouds.

Trends in erythemal irradiance at the Earth’s surface over the period of 2005–2017 have been calculated for the continental United States using satellite-based (OMI) measurements and ground-based measurements at 31 sites distributed throughout the United States by the Department of Agriculture’s UV-B Monitoring and Research Program [241]. The study concluded that trends in noontime erythemal irradiance estimated from these satellite- and ground-based measurements cannot be reconciled. Specifically, trends derived from the satellite-based dataset were not significant for most of the continental United States, except for a small region in the New England states of Maine, Vermont, New Hampshire, and Massachusetts. In those regions, small (about 5% per decade) positive trends were calculated from OMI data, and they were significant at the 95% CL. However, data from the two ground-based stations located in this region indicated a significant decrease in erythemal UV over the same period. This discrepancy can be explained, either by calibration issues of the ground-based sensors and OMI [247], or by increasing attenuation of UV radiation in the lowest part of the atmosphere, which cannot be adequately probed by OMI. While trends calculated for several other stations were also significant, the magnitude of these trends is generally within the measurement uncertainty range so that no firm conclusions about changes in levels of erythemal irradiance across the continental United States can be drawn.

In a similar study based on OMI measurements, trends in noontime erythemal irradiance, TCO, and cloud and haze transmission were calculated for 191 cities located between latitudes of 60° N and 60° S over the period 2005 − 2018 [248]. Significant changes in erythemal irradiance were found at the 95% CL for 40 of the 191 sites over this period. When data were averaged over 15° latitude bands, correlations between erythemal irradiance and short- and long-term changes in cloud and absorbing aerosols, as well as inverse correlations between UV radiation and TCO, became apparent. Estimates of changes in atmospheric transmission at 340 nm show increases of 1.1 ± 1.2% per decade between 60° S and 45°  S, almost no change between 45° S and 20°  N, decreases of 3% per decade at 22° and 32° N, an increase of 2.5% per decade near 25° N, and increases of 1 ± 0.9% per decade from 35° N to 60°  N. Changes in zonally averaged (~ 15° latitude bins) erythemal irradiance between 60° N and 60° S range between –4 and 5% per decade and are predominantly caused by changes in cloud and aerosol transmission. However, judging from the error bars in the figures provided by Herman et al. [248], changes in zonally averaged transmission and erythemal irradiance are generally not significant at the 95% CL.

Trends in erythemal daily doses (D_ery_) were calculated for the period 1979–2015 over Northern Eurasia (a region between 40° and 80° N and extending in longitude from Scandinavia to Siberia) using simulations by a climate chemistry model (INM-RSHU^34^), re-analyses of atmospheric data (ERA-Interim^35^), and data from satellite measurements (TOMS/OMI) [242]. For cloud-free conditions, statistically significant increases in D_ery_ of up to 3% per decade were found for spring and summer over large areas and attributed to decreases in TCO. When clouds were included in the analysis, greater trends of 6–8% per decade were found over Eastern Europe and several regions in Siberia and Northeast Asia. This observation suggests that over this 36-year period, changes in cloud attenuation had larger effects on UV-B radiation than changes in TCO at high latitudes of the Northern Hemisphere extending to 80° N.

An analysis of UVI data computed from satellite-based measurements for local noon and clear skies by the Tropospheric Emission Monitoring Internet Service (TEMIS) indicated that there is no long-term trend in UVI at the equatorial high-altitude site of Quito, Ecuador (0° S, 2850 m asl), for the period 1979–2018 [238]. This conclusion was corroborated by ground-based measurements at this site. For 2010–2014, the measured UVI was within the range of variability inferred for 1979–2009 from TEMIS data. This is consistent with the observation that there are no significant trends in TCO in the tropics (Sect. 3.1).

Trends in the UVI measured by spectroradiometers at three Antarctic sites (South Pole (90° S), Arrival Heights (78°S), and Palmer Station (65°S)) have recently been reassessed for the period of 1996–2018 [190]. At the South Pole (a site representative of the Antarctic Polar Plateau), significant (95% CL) decadal trends of − 3.9% and − 3.1% were calculated for January and February, respectively, which can mostly be explained by concomitant trends in TCO. At Arrival Heights, the recalculated trend for summer is − 3.3% per decade and is significant at the 90% CL. This downward trend is caused by a significant upward trend in TCO of 1.5% per decade for January plus the effect of reductions in land-fast ice covering the sea adjacent to the instrument site (Sec 6.2). No significant trends were reported for Palmer Station. The study provides further evidence that the UVI in Antarctica is starting to decrease during summer months. However, statistically significant reductions for spring (October and November), when the ozone hole leads to large UVI variability, were not detected.

All studies summarized above paint a consistent picture: changes in UV-B radiation outside the polar regions over the last 2–3 decades are mainly governed by variations in clouds, aerosols, and surface reflectivity (for snow- or ice-covered areas), while changes in TCO are less important. These results corroborate the conclusion by McKenzie et al. [56] discussed in Sect. 4.1 that changes in TCO have not led to significant changes in UV-B radiation over this period.

### Reconstruction of historical changes in UV radiation

Systematic measurements of surface UV radiation suitable for trend analysis began only in the late 1980s. In the absence of direct measurements, knowledge of UV irradiance levels prior to the onset of ozone depletion relies on radiative transfer model calculations in combination with inputs such as TCO and other proxy data. At very few locations, ground-based TCO measurements commenced before the 1960s and UV irradiances have been reconstructed from these measurements [249–252]. The erythemal UV irradiance was recently reconstructed for Moscow, Russia, for the warm season (May–September) over the period 1968–2016 [253] using data of TCO, AOD at 550 nm, surface albedo, cloud cover, and cloud transmission. Results were validated against measurements of broadband instruments emulating the erythemal response of human skin (Sect. 10.1), which were available from 1999 onward. Reconstructed and measured data for the overlap period agreed well; the coefficient of determination *R*^2^ was 0.89. Results indicate statistically significant decadal trends in erythemal UV irradiance of –11.6 ± 1.6% for the period 1968–1978 and 5.1 ± 1.9 for the period 1979–2016, which were predominantly driven by changes in cloud transmission. One important shortcoming of the study is that the consistency of cloud data of this 48-year data record was not independently verified; hence, trend estimates could be affected by spurious trends in the measures of cloudiness.

Daily erythemal UV doses were reconstructed for Novi Sad, Serbia [254]. Using a radiative transfer model with inputs of TCO and snow cover data, plus empirical relations between erythemal doses and sunshine duration, statistically significant increases in erythemal UV doses of 8.8% and 13.1% per decade over the period 1980–1997 were found for summer and winter, respectively, which were linked to the statistically significant decline in TCO over this period.

Satellite measurements of TCO became available in the late 1970s and have also been used for reconstructing the UVI at several ground stations under the assumption that changes in aerosol and clouds were small during this period [56]. These reconstructions imply that considerable increases in the summer UVI occurred between 1978 and 1990, ranging from about 5% at northern mid-latitudes, up to 10% at southern mid-latitudes, and up to 20% at the three Antarctic sites considered in this study.

**Fig. 9:**
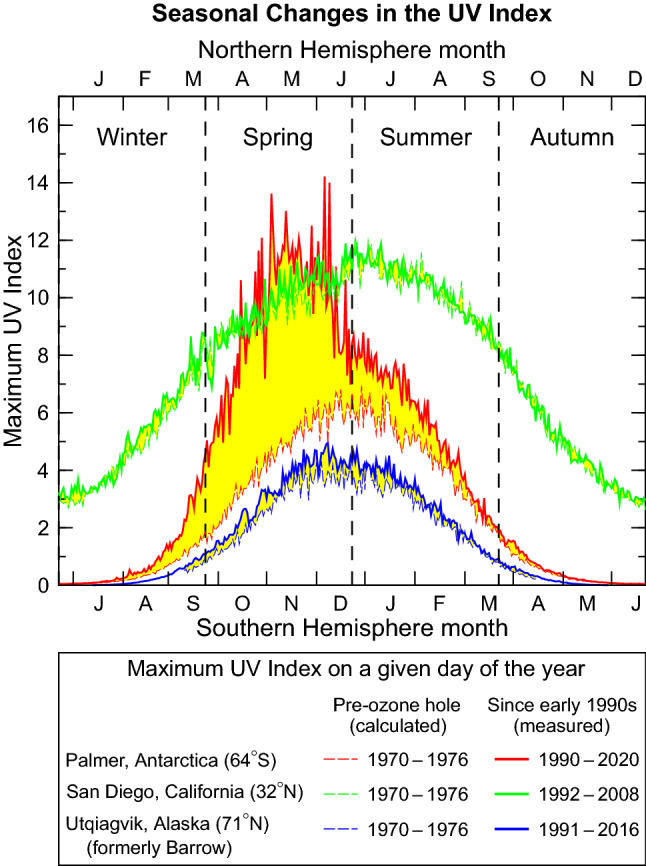
Comparison of the highest UVIs ever measured for each day of the year at Palmer Station, San Diego, and Barrow since the early 1990s (solid lines) with reconstructed data for the pre-ozone-hole period 1970–1976 (broken lines). Yellow shading indicates the change between historical and contemporary UVI. The difference is particularly large for Palmer Station during spring, the period affected by the Antarctic ozone hole. The highest UVIs observed at Palmer since the 1990s exceed those measured at San Diego despite that city’s much lower latitude. Reprinted from Bernhard et al. [230]

Starting in 2010, the “Twenty Questions and Answers About the Ozone Layer” component of assessment reports prepared by the SAP have included a plot comparing reconstructed UVIs at Palmer Station, Antarctica (64° S), for the pre-ozone-hole period 1978–1980 with UVIs measured between 1990 and 2006 [13, 255, 256]. This plot has recently been updated [230] and is reproduced in Fig. 9. The revised plot is similar to the legacy one but includes data up to 2020 and also compares recent measurements with reconstructed pre-ozone-hole UVIs for San Diego, California (32° N), and Barrow, Alaska (79° N). Furthermore, historical UVIs at the three sites have been calculated from TCO measurements by the Backscatter Ultraviolet (BUV) experiment on the Nimbus-4 satellite between 1970 and 1976. While trends in TCO were already negative in the 1970s over polar regions [67], analysis presented by Bernhard et al. [230] did not show clear evidence that the developing ozone hole affected Palmer Station before 1976. In contrast, the period 1978–1980 used for the legacy plot was already somewhat influenced by ozone depletion. The new results confirm the previous conclusion that the ozone hole led to large increases in the UVI at Palmer Station year-round, with the largest increases occurring during spring (between 15 September and 15 November). The maximum UVI at this site is now larger by a factor of 2.50 ± 0.37 (± 1σ) on average compared to the pre-ozone-hole period. During summer and autumn (21 December–21 June), i.e., the seasons least affected by the ozone hole, UVI maxima measured between 1990 and 2020 exceed maxima estimated for years prior to 1976 by 20 ± 13%. Measured and reconstructed pre-ozone depletion data for San Diego (a subtropical site), are almost indistinguishable: on average, the UVI has increased by 3 ± 7% (± 1*σ*) since the 1970s. This modest growth is consistent with the small change in TCO observed at subtropical latitudes (Sect. 3.1) and with the conclusion of McKenzie et al. [56] that maximum daily UVI values have remained essentially constant at mid-latitudes over the last ~ 20 years due to the phase-out of ODSs controlled by the Montreal Protocol. At the Arctic site of Barrow, the UVI increased by 18 ± 15% (± 1*σ*) since the 1970s. The largest spikes in the UVI of up to 40% relative to the 1970s were measured during spring in years with abnormally strong Arctic ozone depletion, such as 2011 [257]. We note that these reconstructions are subject to uncertainty because they assume that surface albedo and attenuation by clouds and aerosols have not changed over the last 50 years in this area. However, at Palmer and Barrow, the TCO is by far the most important factor in controlling the UVI, while changes in albedo at San Diego can be considered negligible. Note that changes in the UVI discussed here do not contradict the conclusion in Sect. 7.2 that long-term changes in UV-B radiation outside the polar regions have generally been small over the last 2–3 decades. Changes shown in Fig. 9 are by and large attributable to changes in TCO occurring in the 1970s and 1980s (Fig. 1).

At Athens, Greece, records of the duration of sunshine were used to reconstruct monthly averages of short-wave (wavelength range ~ 300–3000 nm) solar irradiance at the surface between 1900 and 2012 [258]. There were very small (0.02%) changes between 1900 and 1953, followed by a negative trend of 2% per decade during a “dimming” period of 1955–1980 and a positive trend of 1.5% per decade during a “brightening” period of 1980–2012. Measurements of short-wave irradiance at Potsdam (52°), Germany, show distinct dimming and brightening periods between 1947 and 1986 and 1986–2016, respectively, with measurements in 1986 about 10 W/m^2^ lower compared to those at the start and end of the time series [259]. Changes for “all-sky” (cloudy and cloud-free) and clear-sky (cloud-free) conditions were similar, suggesting that changes in aerosols were mostly responsible for these variations in short-wave irradiance. While these trend estimates are unrelated to changes in TCO and do not directly translate to changes in UV radiation, they qualitatively capture variations in the effect of clouds and aerosols on solar irradiance over the period studied, which are also relevant for changes in UV radiation. Trends in UV radiation related to changes in aerosols are likely larger than trends in short-wave irradiance because attenuation by aerosols is generally larger in the UV region than at visible wavelengths.

## Projections of UV radiation

Projections of solar UV radiation at the Earth’s surface for the twenty-first century have been reported in new studies published during the last four years. These new projections are generally in agreement with those reported in our last assessment [9]. They confirm the projected reductions in UV radiation, particularly at high and polar latitudes, due to the recovery of stratospheric ozone, as well as the increases in UV radiation due to decreasing concentrations of aerosols over regions with intense urban or industrial activities. Furthermore, projected decreases in surface reflectivity due to reduction in ice cover and decreases in cloudiness, both associated with climate change, are also important drivers leading to regional changes (decreases and increases, respectively) in surface UV radiation.

One of the new studies [260] reports global projections of UVI that were calculated with a radiative transfer model using TCO, temperature, and aerosol fields provided by 17 CCMs. These CCMs were included in the first phase of the Chemistry-Climate Model Initiative (CCMI-1) [58, 261]. The CCM simulations were performed for four future GHG scenarios described by RCPs 2.6, 4.5, 6.0 and 8.5. Zonal-mean noontime UVI for cloudless skies were calculated for the period 1960–2100.

According to this study, noontime UVI in 2100 is projected to increase relative to calculations for the 1960s for RCPs 2.6, 4.5 and 6.0. These increases depend on latitude and the RCP scenario, and range between 1% (northern high latitudes for RCP 6.0) and 8% (northern mid-latitudes for RCP 2.6) as shown in Table 1. Trends calculated for the worst-case scenario RCP 8.5 show a different pattern with UVI projected to increase only in the tropics and to decrease elsewhere, with the largest decrease of 8% at northern high latitudes.

**Table 1:** Comparison of zonal mean changes in clear-sky UVI calculated by Lamy et al. [260] and Bais et al. [9]

Latitudes	Lamy et al. [260]							Bais et al. [9]			
	Change [%] 1960 to 2100^a^					Change [%] 2015 to 2090^b^		Change [%] 2015 to 2090^c^			
	Transient AODs, RCP =				Fixed AODs	Transient AODs	Fixed AODs	Fixed AODs			
	2.6	4.5	6.0	8.5	RCP 6.0	RCP 6.0	RCP 6.0	RCP 6.0			
	Annual mean^e^							Jan	Apr	Jul	Oct
High N^d^	6	2	1	− 8	– 5	0	– 6	– 3	– 7	– 5	– 4
30–60° N	8	5	5	– 1	– 2	5	– 3	– 4	– 5	– 3	– 2
0–30° N^f^	3	3	3	1	3	–	1	– 1	0	– 1	– 1
0–30° S^f^	3	3	3	2	3	0	0	– 1	0	– 1	– 1
30–60° S	3	3	2	– 2	0	– 5	– 6	– 5	– 4	– 5	– 6
High S^d^	7	6	4	0	– 2	– 18	– 18	– 8	– 6	– 6	– 23

All values are rounded

^a^According to Table 5 of Lamy et al. [260]

^b^Inferred from Figs. 4, 6 of Lamy et al. [260]

^c^Table 1 of Bais et al. [9]

^d^Latitude range of “High N” and “High S” refer to 60–90° in Lamy et al. [260] and 60–80° in Bais et al. [9]

^e^Changes reported by Lamy et al. [260] refer to trends averaged over all months; Bais et al. [9] provide changes for the months of January, April, July, and October

^f^Lamy et al. [260] report changes separately for 0–30° N and 0–30° S while Bais et al. [9] provide changes for 30° N to 30° S

Only three of the 17 CCMs provided outputs of the AOD and its wavelength-dependence. The AOD used in projections of UV radiation is therefore based on the median of AODs derived from these three CCMs. According to these calculations, AODs are projected to decrease by almost 80% between 2000 and 2100 at northern high- and mid-latitudes, resulting in concomitant increases in the UVI of about 2% and 6%, respectively. These changes in UVI due to changes in AOD are of similar magnitude to those caused by changes in stratospheric ozone. However, these AOD estimates as well as the absorption properties of aerosols used in these CCMs are highly uncertain because future changes in atmospheric aerosols depend greatly on policy choices, such as measures to reduce air pollution [262]. Moreover, changes in optical depth and absorption properties of aerosols are highly dependent on region, hence zonal mean changes in UVI, like those discussed above, are not necessarily representative for most regions.

To address these concerns, Lamy et al. [260] also provide UVI projections for temporally invariant or “fixed” AODs based on a current climatology [263]. Using this climatology and the RCP 6.0 scenario, noontime UVIs in 2100 are projected to change relative to 1960 by –5% at Northern Hemisphere (NH) high latitudes, –2% at NH mid-latitudes, + 3% in the tropical belt, 0% at Southern Hemisphere (SH) mid-latitudes, and –2% at SH high latitudes (Table 1, column 6). These changes in UVI are mainly driven by changes in TCO. Assuming time-invariant aerosol amounts for the future, the clear-sky UVI is projected to decrease from 2015 to 2090 by 3% at NH and 6% at SH mid-latitudes. However, in regions that are currently affected by air pollution, the UVI is projected to increase if emissions of air pollutants are curtailed in the future.

Table 1 also shows a comparison of projected changes in zonal mean UVIs between 2015 and 2090 inferred from the study by Lamy et al. [260] and as published in our last assessment [9]. In both cases, UVI projections were based on results from the CCMI-1 initiative; however, different subsets of models were used, as well as methods to calculate the UVI from parameters provided by the CCMs. Furthermore, Bais et al. [9] provided projections for different months while Lamy et al. [260] only considered annual averages. Despite these differences, changes in UVI calculated by the two studies for fixed AODs are consistent (see last five columns of Table 1) and project a decrease of 2–5% for northern mid-latitudes, a decrease of 4–6% for southern mid-latitudes, and almost no change for the tropics. Both studies also predict large decreases in the UVI over southern high latitudes due to the expected healing of the stratospheric ozone hole.

Projections provided in the above studies were corroborated by another study where long-term changes in erythemal UV radiation were calculated over Eurasia (latitudes 40–80° N, longitudes 10° W–180° E) based on results of a CCM developed by the Russian State Hydrometeorological University (RSHU) [264]. These calculations considered only changes in TCO (i.e., excluding effects of aerosols and clouds) and predict that erythemal UV radiation levels in the years 2055–2059 will be lower over Eurasia by 4 to 8% relative to the reference period 1979–1983.

Simulations with one of the CCMs (EMAC^36^) for the period 1960–2100 were used to derive trends in DNA-damaging radiation at four mid-latitude locations and one tropical high-altitude site [265]. Weighting the spectral irradiance with the action spectrum for DNA-damage [266] yields dose rates that are more sensitive to changes in radiation at shorter (UV-B) wavelengths than the erythemal UV dose rates or the UVI; hence it is more sensitive to changes in TCO. DNA-damaging irradiance averaged over the five locations considered in this study is projected to increase by 1.3% per decade between 2050 and 2100. To isolate the effect of GHGs on climate, one simulation assumed increasing GHGs according to RCP 6.0 and the second adopted constant GHGs at 1960 levels. No trend in TCO was detected by the model after 2050, and the trend detected in DNA-damaging irradiance was attributed to a statistically significant (95% CL) decrease in cloud cover of 1.4% per decade resulting from increasing GHGs. The study suggests that changes in UV-B irradiance at low- and mid-latitudes during the second half of the twenty-first century will be dominated by factors other than changes in stratospheric ozone. However, these projections depend on the accuracy of simulating the cloud fields by climate models because uncertainties in the modeling of clouds propagate to the projected changes in solar UV-B radiation.

The SAP’s latest assessment [11] also evaluates the effect of a fleet of commercial supersonic aircraft on stratospheric ozone concentrations. Such a fleet is currently being considered by different organizations and companies. Depending on scenario and flight altitudes, emissions of water vapor and nitrogen oxides from such a fleet could reduce TCO by up to 25 DU at high northern latitudes. Reductions in TCO at mid and low latitudes of the Northern Hemisphere would be considerably smaller, and the Southern Hemisphere is less affected because most flights take place in the Northern Hemisphere. While no study has quantified the effect of a future fleet of supersonic aircraft on UV radiation, the estimated decrease in TCO suggests that erythemal UV irradiance could increase by several percent at mid-latitudes of the Northern Hemisphere.

New model calculations examined the effect on stratospheric ozone (and by implication on UV radiation) of quadrupling concentrations of atmospheric CO_2_ [267]. Such an increase would lead to a dynamically-driven decrease in concentrations of ozone in the tropical lower stratosphere, an increase of ozone in the lower stratosphere over the high latitudes, and a chemically driven increase of ozone (via stratospheric cooling) throughout the upper stratosphere. In the tropics, opposite changes in ozone in the upper and lower stratosphere result in small changes in the TCO, and, in turn, to small changes in tropical UV-B radiation in the future, if effects from all other factors remain the same. A quadrupling of atmospheric CO_2_ concentrations during the twenty-first century is currently not expected, but could occur in the twenty-second century if emissions of CO_2_ were to continue unabated according to the RCP 8.5 scenario [268]. The study suggests that even “worst-case” increases in CO_2_ will not result in significant increases in UV-B radiation in the tropics.

All studies discussed above confirm the understanding of UV radiation in the twenty-first century established in our last assessment [9]. Simulations with a new generation of CCMs that have only recently been performed are expected to provide updated projections of UV radiation but are not yet available for assessment in this report.

A recent study used a state-of-the-art climate model with interactive chemistry [269] to calculate the effects on TCO and UV radiation resulting from a regional or global nuclear war. A global-scale nuclear war would cause a 15-year-long reduction in the TCO with a peak loss of 75% globally and 65% in the tropics. Initially, soot would shield the surface from UV-B radiation, but eventually the UVI would become extreme: greater than 35 in the tropics for 4 years, and greater than 45 during the summer in the southern polar regions for 3 years. For a regional nuclear war, global TCO could be reduced by 25% with recovery taking 12 years.

## Implications of solar radiation management on UV radiation

Over the last decade, global warming from increasing GHGs has accelerated, and global mean air temperatures near the surface have risen by about 1.1 °C above pre-industrial levels [Chapter 2 of 63]. The resulting changes in climate observed worldwide have stimulated discussions on strategies to mitigate warming through artificially forced reduction of solar radiation entering the troposphere. Impacts of such solar radiation management (SRM) interventions on the atmosphere and the environment have been investigated in numerous modeling studies and discussed in current assessments by the SAP [11] and IPCC [Chapter 4 of 63], and the last EEAP assessment [9]. The latest SAP report [11] extensively addresses the potential impacts on TCO from stratospheric aerosol injection (SAI) under different scenarios. Here, we focus on the effects of the possible implementation of SAI on surface UV radiation. The effects are driven not only by changes in TCO but also by the redistribution of solar radiation from the direct-to-diffuse component, plus the global dimming effect expected from back-scattering of solar radiation to space by the aerosol layer.

The TCO is affected both by SAI-induced changes in heterogeneous chemical reactions, which depend on the surface area density of the aerosol (e.g., in µm^2^/cm^3^), and by changes in atmospheric dynamics (including transport, temperature, and water vapor changes). These effects on TCO differ with latitude and season, and depend on the future SAI scenario because they act in addition to the effects of decreasing ODSs and increasing GHGs. During the Antarctic ozone hole season, destruction of ozone in the stratosphere resulting from SAI would mainly be controlled by halogen chemistry on the surface of aerosols, while transport of ozone through circulation becomes important in other seasons [11].

Using models that participated in the Geoengineering Large ENSemble (GLENS) project, Tilmes et al. [270] estimated the effect on TCO in the latitude band 63°–90° S from SAI designed to achieve a reduction of 1.5 and 2.0 °C in global surface temperature. They found a reduction of up to 70 DU in the Antarctic TCO at the start of the SAI application (2020–2030), followed by an increase in TCO towards 2100 with a pattern like the projected changes in TCO without the application of SAI. In a more recent study, Tilmes et al. [271] estimated the initial abrupt decrease in TCO to be between 8 and 20% in 2030–2039 compared to 2010–2019, depending on injection strategy and model. All scenarios assumed in these studies result in a delayed recovery of Antarctic ozone to pre-ozone-hole levels by 20 to ~ 40 years. The TCO for these SAI scenarios remains below the levels projected by the worst case GHG scenario (SSP5-8.5) until the end of the twenty-first century, which would lead to increased levels of UV-B radiation during the entire period in Antarctica.

In a similar study, Tilmes et al. [272] estimated the effects of SAI also in the Northern Hemisphere and the tropics based on simulations of the G6 Geoengineering Model Intercomparison Project (GeoMIP). The models agree that sulfur injections result in a robust increase in TCO in winter at middle and high latitudes of the Northern Hemisphere of up to 20 DU over the twenty-first century compared to simulations based on the SSP5-8.5 scenario without SAI. This increase in TCO, which is linearly related to the increase in the amount of sulfur injections, is driven by the warming of the tropical lower stratosphere and would eventually result in decreasing UV-B radiation at these latitudes during the remainder of the twenty-first century. The magnitude of these changes in UV-B radiation depends on the SAI scenario. The Arctic TCO is initially projected to decrease by 13 to 22 DU depending on the scenario, which is a much smaller decrease than that projected by Tilmes et al. [270] for the Antarctic discussed above. By the end of the twenty-first century, the Arctic TCO with and without SAI are approximately the same. Finally for the tropics, changes in ozone due to SAI would be small. The initial reduction in TCO projected by Tilmes et al. [270] and Tilmes et al. [272] for the Antarctic and Arctic is attributable to heterogeneous reactions on aerosol particles in the presence of ODSs. Robrecht et al. [273] showed that this effect is far less important for mid-latitudes and the tropics compared with polar regions.

While the above studies have focused on the consequences of SAI on ozone, effects on UV and visible radiation from SAI also depend on the attenuation (dimming) and redistribution of solar radiation. These effects have been quantified with a radiative transfer model using inputs from the GLENS project [271] designed to counteract warming from increased GHGs under the RCP 8.5 scenario [274]. Estimated changes in the UVI are predominantly driven by the attenuation of solar radiation by the artificial aerosol layer (with concentrations peaking above ~ 30 km in the tropics and above ~ 25 km in the high latitudes). Reduced direct radiation due to aerosol scattering results in substantial reductions in solar irradiance at the Earth’s surface despite an enhanced contribution from diffuse radiation. However, the larger diffuse component may allow more efficient penetration of UV irradiance through forest and crop canopies [275], offsetting, to some extent, the reduced irradiance on top of the canopies. The intervention is estimated to reduce the daily average above-canopy UVI in 2080 relative to 2020 by about 15% at 30° N and by 6–22% at 70° N, depending on season. About one third of the reduced UVI at 30° N is due to the relative increase in TCO (~ 3.5%) between the reference and the SRM scenario. The corresponding increase in TCO for 70° N is less than 1% and explains only a very small fraction of the decrease in the UVI. The calculated changes in the UVI are therefore primarily caused by the scattering effect of sulfate aerosols, with a very small contribution from the absorption by sulfur dioxide (SO_2_). Finally, reductions in photosynthetically active radiation (PAR) are estimated to range from 9 to 16% at 30° N and from 20 to 72% at 70° N, depending on season, with the largest proportional changes occurring in December, when the absolute levels of radiation are small. Such large changes in the UVI and PAR would likely have important consequences for ecosystem services and food security; however, such repercussions have not yet been quantified. While the study only characterized changes in UV radiation and PAR for the NH, similar results can be expected for the SH.

## Advances in UV monitoring and modeling

In this section, we provide a summary of advances in measuring and modeling UV radiation at the Earth’s surface and in assessing personal exposure to UV radiation, which is controlled both by ambient UV radiation and personal behavior.

### Ground-based systems

UV radiation at the Earth’s surface is normally measured with scanning spectroradiometers, such as those installed in the Network for the Detection Atmospheric Composition Change (NDACC) [276]; broadband instruments, which typically emulate the erythemal response of human skin [277]; multi-filter instruments, which measure the spectral irradiance at several wavelengths (typically 4–7) in the UV range [278]; array spectroradiometers, which record the entire UV spectrum within seconds; dosimeters, which measure the UV dose that accumulates over a given amount of time; and specialized systems designed for a specific research question such as the measurement of the angular distribution of sky radiance [279]. The different instruments have been discussed in detail in previous assessments [9, 162]. In brief, scanning spectroradiometers using double monochromators are the most accurate instruments but are expensive to acquire and maintain, and the recording of a UV spectrum may take several minutes. Broadband radiometers are relatively inexpensive, and their spectral response is tailored to a specific effect (e.g., erythema) under study, but because they do not provide spectral information, the factors driving changes in UV radiation (e.g., ozone, clouds, and aerosols) cannot be unambiguously separated. Multi-filter instruments can be used for studying a specific effect and the factors it depends upon, but require elaborate characterisations and calibrations to provide accurate data of solar irradiance [280]. Array spectroradiometers (or spectrographs) use single monochromators for physical reasons, and measurements at wavelengths shorter than 310 nm are often affected by stray light [281]. An instrument combining an array spectrometer with narrow-band filters that mitigate this problem has recently been introduced [282] and evaluated by [283], indicating good performance at wavelengths longer than 305 nm. Finally, dosimeters are simple, low-cost, small devices that measure the UV dose electronically [284], chemically [285, 286], or both [287], and are further discussed in Sect. 10.5.2. Their accuracy is typically less than that of high-end spectrometers [288]; however, they are frequently used for exposure studies (Sect. 10.5.2) where they can be easily attached, for example, to the forehead, wrist or clothing of test subjects.

The quality of measurements of UV radiation from these systems or sensors has historically been assessed with intercomparison campaigns where instruments are either compared with each other or a reference instrument. An example of the latter is a campaign with 75 participating broadband radiometers with erythemal response [277]. The instruments’ solar measurements were first compared with data from the QASUME (Quality Assurance of Spectral Ultraviolet Measurements in Europe) reference spectroradiometer [289]. The QASUME instrument has been used since 2002 to assess the quality of UV radiation measurements from more than 250 spectroradiometers at more than 40 stations worldwide (https://www.pmodwrc.ch/en/world-radiation-center-2/wcc-uv/qasume-site-audits/). New calibrations were subsequently transferred from QASUME to the 75 broadband radiometers. Furthermore, the angular and spectral response of the instruments was measured and functions for correcting deviations from the ideal response were established. With their original calibration applied, measurements of 32 (43%) of the 75 instruments agreed to within ± 5% with measurements of the reference spectroradiometer while 48 (64%) agreed to within ± 10%. Twenty-seven (35%) datasets deviated by more than ± 10% from the reference and two datasets differed by 70%. After instruments were recalibrated, 73 (97%) of the 75 instruments agreed to within ± 5% with the reference. This example demonstrates that proper quality control, quality assurance, and calibration procedures are vital for obtaining accurate measurements of UV radiation. A similar intercomparison involving four broadband radiometers and a reference spectroradiometer was conducted between March 2018 and February 2019 at Saint-Denis, La Réunion (21° S) [290]. Data from three of the four instruments agreed to within ± 3% with the reference while data from one instrument exhibited a systematic error of 14%.

Even high-end spectroradiometers require meticulous characterization and calibration for obtaining measurements with low uncertainty [291]. Finally, the development of a rigorous uncertainty budget (i.e., the calculation, tallying and combination of all uncertainty components) is a demanding task [292], but is necessary for obtaining high quality data.

### Modeling of UV radiation

The transfer of radiation through the Earth’s atmosphere is affected by absorption and scattering by gases, aerosols, and clouds; the reflection of radiation by the Earth’s surface; and several other factors (Sect. 6). These factors are taken into account in computer simulations of UV radiation by radiative transfer models. Physically correct radiative transfer codes for modeling the UV radiation at the Earth’s surface have been available for many years [e.g., 293,294–297] and can be considered reliable and mature. Most models assume that the atmosphere is homogeneous in both horizontal directions and only varies in the vertical direction, but newer models (e.g., [298, 299]) that are based on the Monte Carlo technique [300] can also account for the three-dimensional structure of the atmosphere, topography, surface condition (e.g., patchy snow) or illumination geometry (e.g., the inhomogeneous irradiation during a solar eclipse). The greatest challenge in radiative transfer calculations is not the physical description of the transfer of radiation through the atmosphere but the specification of the input parameters that interact with radiation and are often not completely known, such as the single scattering albedo (SSA) of aerosols in the UV-B range or the structure of clouds.

One source of uncertainty in determining the UV radiation at the Earth’s surface with models is the uncertainty of the solar spectrum outside the Earth’s atmosphere. The extraterrestrial solar spectra (ETS) used in legacy model implementations sometimes differed by several percent at certain wavelengths [301, 302]. These surprisingly large discrepancies for a fundamental quantity such as the ETS can be explained by the difficulty in measuring this spectrum. In one method, several solar spectra are observed at the Earth’s surface at different path lengths of the direct solar beam through the atmosphere. These measurements are then extrapolated using the Langley technique [303] to a path length of zero for deriving the ETS. The method is subject to large uncertainties at wavelengths where atmospheric attenuation is large, such as at wavelengths shorter than 310 nm (where ozone absorbs strongly) or in strong water vapor absorption bands. Another method is the direct measurement from space. The challenge of this method is to prevent changes in an instrument’s calibration during transport from the calibration laboratory to space. Both methods have advanced greatly during the last years.

Gröbner et al. [304] applied the Langley technique to radiometrically accurate measurements of QASUME (Sec 10.1) and a “Fourier-transform spectroradiometer,” which measures spectra at high resolution, to derive an ETS over the wavelength range of 300–500 nm with a spectral resolution of 0.025 nm, a wavelength accuracy of 0.01 nm, and a radiometric accuracy of 2% (95% CL) between 310 and 500 nm and 4% at 300 nm. Richard et al. [305] measured the ETS from the International Space Station with the Total and Spectral Solar Irradiance Sensor / Spectral Irradiance Monitor (TSIS-1 SIM) between 200 and 2,400 nm with an accuracy of 0.5% (95% CL) and a spectral resolution of 5 nm between 280 and 400 nm. The high accuracy is achieved by calibrating the system against a cryogenic radiometer and monitoring the instrument’s stability in space with an on-board, detector-based reference electrical substitution radiometer. Finally, by combining the superior spectral resolution of the spectrum by Gröbner et al. [304] with the greater radiometric accuracy of the TSIS-1 SIM spectrum, Coddington et al. [306] developed a composite spectrum (named TSIS-1 HSRS) with a spectral resolution of 0.025, a sampling resolution of 0.01 nm and a radiometric accuracy of better than 1.3% (68% CL) at wavelengths shorter than 400 nm, representative of solar minimum conditions between solar cycles 24 and 25. This spectrum can be considered a new benchmark for modeling applications.

An important application of radiative transfer models is the calculation of UV irradiances at the Earth’s surface from backscattered radiances measured by satellites (Sect. 10.3). Typically, measurements at different wavelengths by a single space-based instrument such as OMI are used to first derive the TCO and then apply corrections to account for the effects of clouds and aerosols [307].

### Satellite observations of UV radiation

The TCO and UV radiation at the ground have been estimated from measurements of various space-borne sensors since the 1970s, starting with the Backscatter Ultraviolet (BUV) experiment on the Nimbus-4 satellite [308]. These measurements have been continued, amongst others, by several Solar Backscatter UV (SBUV) instruments [309]; Total Ozone Monitoring Spectrometers (TOMS) [310, 311]; Global Ozone Monitoring Experiments (GOME and GOME-2) [312, 313]; the Ozone Monitoring Instrument (OMI) [314] on the Aura satellite; and the Earth Polychromatic Imaging Camera (EPIC) installed on the Deep Space Climate Observatory (DSCOVR), which is located at the Lagrange Point L1 between the Earth and Sun [315].

Several of these types of instruments have been installed on various satellites. Estimates of UV radiation are derived from backscattered radiances measured by these sensors and radiative transfer model calculations (Sect. 10.2). Uncertainties of these estimates are typically larger than those of UV measurements at the Earth’s surface because the conditions on the ground cannot be completely characterized from space, in particular in the presence of clouds [316], absorbing aerosols in the boundary layer [317], or snow and ice [318]. The validation of satellite data with ground-based measurements from many sites has been discussed in our previous assessment [9]. In general, UV data from satellites are accurate within a few percent under low-aerosol and clear-sky conditions, but can be affected by systematic errors exceeding 50% for less ideal observing conditions.

Data of UV radiation at the Earth’s surface estimated from satellite observations typically have the spatial resolution of the satellite sensor (e.g., 13 × 24 km at nadir for OMI) and are typically based on one satellite-measured spectrum per day at low and mid-latitudes. As an alternative, Kosmopoulos et al. [319] have used inputs from various data sources to calculate real time and forecasted UVIs for Europe with a spatial and temporal resolution of 5 km and 15 min, respectively. The new data product agrees with measurements at 17 ground-based stations distributed across Europe to within ± 0.5 UVI units for 80% of clear-sky and 70% of all-sky conditions. Similarly, Vuilleumier et al. [320] calculated erythemal irradiance for Switzerland with a spatial resolution of 1.5–2 km and a temporal resolution of one hour for 2004–2018, using data from several European satellites. A validation of these data with ground-based measurements at three meteorological stations in Switzerland (Locarno, Payerne, and Davos) indicates that the expanded uncertainty of hourly UVI values of the new data products is about 0.3 UVI units for UVI < 3 and up to 1.5 UVI units for UVI > 6.

Measurements with OMI started in 2004 and their quality has degraded recently [247]. The future of the Aura spacecraft is uncertain beyond 2023 [321]. Fortunately, several alternative satellite instruments have become operational within the last years to continue monitoring of ozone and UV radiation from space. For example, the Ozone Mapping and Profiler Suite (OMPS) [322] is installed on NOAA’s Suomi NPP (launched in 2011) and the NOAA-20 (launched in 2017) satellites. The TROPOspheric Monitoring Instrument (TROPOMI) [323], which is installed on the Sentinel-5 Precursor satellite (launched in 2017), will continue ozone-monitoring efforts by the European Space Agency. TROPOMI may also fly on future Sentinel satellites [324]. TROPOMI observations of UV radiation have recently been compared with ground-based measurements at 25 sites [325]. For snow-free surface conditions, the median relative difference between UVI measurements by TROPOMI and these ground stations was within ± 10% at 18 of 25 sites. For 10 sites, the agreement was at the ± 5% level. These differences are comparable to those reported for OMI [316, 318, 326, 327]. Larger differences were observed at locations with challenging conditions, such as mountainous areas or sites in the Arctic and Antarctic with variable snow cover. A comprehensive comparison between OMI and TROPOMI surface UV products is planned [314] to ensure that there is no step-change in the time series of UV radiation measurements when transitioning from OMI to TROPOMI.

In preparation for new satellite missions (e.g., Sentinel-4 and Sentinel-5 of the European Space Agency), Lipponen et al. [328] developed an approach to assimilate input data from geosynchronous and low Earth orbit satellite measurements with the goal to provide high-resolution UVI and UV-A data. Zhao and He [329] combined TCO data from OMI with top-of-the-atmosphere reflectance data from MODIS for quantifying attenuation by clouds and aerosols and surface reflectance data from MODIS and used a machine learning algorithm to calculate erythemal irradiances at 1 km resolution. The system is trained and tested with UV measurements of NOAA’s Surface Radiation Budget Network (SURFRAD) and UV data from the United States Department of Agriculture’s (USDA) UV-B Monitoring and Research Program. For most stations, calculated and measured data agreed to within ± 5% (mean bias calculated from match-up data). However, the system was trained with data from the continental United States only, and the fidelity of the method for sites that are different in terms of latitude, ozone climatology, pollution levels, and surface albedo has not yet been demonstrated.

### Forecasting of the UV Index

The UVI is now part of weather forecasts in many countries. National weather services and other agencies use models to predict the diurnal course of the UVI (e.g., every hour) for one or several days into the future (e.g., the Israel Meteorological Service (https://ims.gov.il/en/UVIHourly), the German Meteorological Service (https://kunden.dwd.de/uvi/index.jsp), and the Copernicus Atmosphere Monitoring Service (https://climate-adapt.eea.europa.eu/observatory/evidence/projections-and-tools/cams-uv-index-forecast). New methods for improving UVI forecasts have recently been proposed based on an “ensemble member” approach, where a model is executed multiple times with different initial conditions [330], and a machine learning algorithm [331].

### Personal exposure

Our 2014 and 2018 assessments [9, 162] discussed advances in the understanding of personal exposure to ambient solar UV radiation and how personal exposure relates to measurements of UV irradiance, which are typically referenced to a horizontal surface. Exposure studies address needs for both research and public advice and quantify UV radiation on non-horizontal surfaces, and how the effects of shade, clothing, and human behavior affect UV doses in real-world settings. Exposure studies have shown that adults working outdoors receive only about 10% of the total available annual UV radiation dose, while indoor-working adults and children get only about 2–4% of the available UV dose [332, 333]. This shows that standard irradiance measurements are a poor proxy for realistic exposures. While there could be a good correlation between ambient and personal UV dose at the population level, exposure of individuals depends greatly on lifestyle. Reviews of a large number of studies on personal exposure to UV radiation during non-occupational [334] and occupational [335] activities concluded that understanding of human exposure to UV radiation has greatly increased during the last 4–5 decades. However, for most activities, our ability to accurately calculate the UV exposure of exposed body sites is still limited for many conditions.

#### Exposure models

Models of human morphology can quantify the protection afforded by attire, for example, from wearing various hats [336] and sunglasses [337]. These models often use the “predictive protection factor” (PPF), which is akin to the sun protection factor (SPF) developed for sunscreens, except that the PPF also depends on the direct-to-diffuse ratio of incident radiation. These models may be validated using mannequin torsos or heads equipped with UV sensors [338]. The sky view factor derived from all-sky imagery in the visible range together with the calculated clear-sky UV irradiance has recently been utilized to accurately estimate UV irradiance in partially shaded settings [339].

Doses of erythemal radiation received by the human body during holidays at the beach have recently been modeled [340]. Taking into account all confounding factors affecting exposure (e.g., clothing, behavior, photo-protection), these models predict that the forearm typically receives about 170 standard erythemal doses (SED) in a week, which is comparable with the average annual exposure of a citizen in Europe or North America. Furthermore, for a full day sun-bathing at the beach or pool, multiple body sites can receive more than 50 SED.

#### Personal dosimetry

The three types of dosimeters previously identified [162]—polysulphone (a plastic film that changes its transmission following exposure to UV radiation), biofilm, and electronic devices—are still in use, and their relative merits in different contexts have recently been reviewed [341, 342]. These measurement technologies were further described in a review that also proposes a future course for development and regulation of wearable UV sensors [343].

Some authors [e.g., 344] distinguish between “radiometers,” which give an instantaneous flux reading such as the UVI, and “dosimeters,” which measure cumulative dose such as the standard erythemal dose (SED). However, the distinction is irrelevant for many electronic sensors, which measure flux but also accumulate it electronically. The same can apply to photochromic sensors in combination with smartphones or other electronic logging. Hereafter, we use the term “dosimeter” for all types of sensors.

The history and characteristics of polysulphone dosimeters have been reviewed by one of their pioneers [285]. They are useful whenever water resistance is necessary, as in a study of triathletes [286]. Alternative photochromic sensors have been developed using the photodegradable dye DTEC^37^ [345] and xanthomattin [344].

A new development of a biofilm dosimeter that mimics the photoreaction resulting in previtamin D_3_ synthesis in human skin has recently been presented [346]. Biofilm sensors of a similar type were used to measure exposure to UV radiation of lifeguards, demonstrating that this group receives high doses of erythemal UV radiation, averaging over 6 SEDs per day [347].

Electronic dosimeters have some advantages for research involving personal dosimetry compared to other sensors. They can be engineered to have a spectral responsivity and a directional response approaching those of research-grade radiometers measuring erythemal irradiance [284]. The time resolution and ability to interface wirelessly with smartphones allows feedback to users, and has supported research on how such information can influence sun exposure amongst melanoma survivors [348], dockworkers and fishermen [349], or young adults in general [350]. In a small study of outdoor workers in Romania, dosimeters measured up to 6 SEDs per day and led the authors to suggest that UV dosimeters should be compulsory for outdoor workers, similar to personal dosimetry for ionizing radiation in relevant professions [351].

A 14-year study with electronic dosimeters showed that participants that are in continued employment maintained their sun exposure behavior, retirees increased their exposure, and high school students reduced their exposure when starting work [352]. Additional exposure studies confirmed expectations that outdoor workers [351]; participants in triathlons [286]; and elite surfers, windsurfers, and Olympic sailors [353] are at high risk of overexposure to UV radiation. In general, staying outdoors for long periods, even at low UV irradiance levels, can result in risk of damage from UV radiation [232].

Airline pilots have long been known to have twice the incidence rate of malignant melanoma and keratinocyte skin cancers than the general population, but UV-B radiation is almost entirely blocked by cockpit windows [354]. Other factors explaining this elevated risk of skin cancer, like ionizing radiation and disrupted circadian rhythms, have been largely ruled out. Measurements with dosimeters that are sensitive to both erythemal and UV-A radiation suggested that cockpit windows are partially transparent to UV-A radiation and pilots are therefore exposed to levels of UV-A radiation that exceed guidelines for eye protection established by ICNIRP [355], in particular if sunglasses are not worn or visors are not deployed [356].

#### Low-cost/crowd-sourced sensors and cell phone apps

Our last assessment [9] described a wide range of new tools for research and for getting information to users, including electronic sensors, photochromic films with associated software, and forecasts or “nowcasts” of UV radiation using cell phone apps. A review of developments in this area [357] describes the promise of these new technologies, but a comparison of UV radiation reported by cell phone apps with actual UV measurements found that many of these apps have poor accuracy [358]. For example, of the six apps reviewed in this study, only one was able to predict the actual UVI to within ± 30% in most cases. A further miniaturization of sensors to millimeter scale with wireless communication to standard consumer devices [359] will widen the scope of how these sensors can be deployed. Other studies have also shown that useful personal exposures to UV radiation can be achieved from satellite-based UV radiation estimates combined with exposure ratio modeling to account for individual factors [360] or by leveraging UV data from local research stations [361].

## Action spectra

Action spectra describe the wavelength dependence of biological effects caused by UV radiation. A biological effect is quantified by first multiplying the action spectrum for this effect by the spectrum of the incident irradiance and then integrating this product over wavelength. The result is the biologically effective UV irradiance, UV_BE_. Most action spectra *decrease* by several orders of magnitude towards longer wavelengths in the UV-B range. Since solar spectra *increase* by a similar amount in this wavelength range, a given biological effect is very sensitive to the wavelength intervals within the UV-B range over which this decrease (action spectrum) or increase (solar spectrum) occurs. This implies that action spectra must be very accurately measured.

The most widely used action spectrum is that for erythema [10], which is the basis of UVI calculations. In sunlight, the strongest contribution to erythema is from UV-B wavelengths, peaking near 307 nm. UV-A wavelengths also contribute, especially at the shorter end of the UV-A region (e.g., 315–340 nm). A small-scale study with 10 participants [362] found clinically perceptible erythema after exposure to UV radiation in the 370–400 nm range plus visible light (400–700 nm), confirming that longer UV-A wavelengths can also cause erythema. The study also suggests that the erythema action spectrum, which is currently defined only up to 400 nm [10], should possibly be extended into the visible range. This finding is also supported by a recent assessment by Diffey and Osterwalder [363].

Another important action spectrum for human health defines the wavelength dependence of the conversion of 7-dehydrocholesterol in the skin to previtamin D_3_, which is subsequently transformed to the active form of vitamin D (1,25-dihydroxycholecalciferol or calcitriol) involving isomerisation and hydroxylations in the skin, liver, and kidneys. This spectrum was measured 40 years ago [364] and was standardized by the International Commission on Illumination (CIE) [365] by interpolating the original data, plus extending the end of the spectrum from 315 to 330 nm via an exponential extrapolation. The spectrum has been widely used for developing recommendations for optimal solar exposure [179]; however, its validity has been questioned [179, 366]. Specifically, the CIE standard [365] is based on a scanned figure from a single publication that does not include a complete description of the experiment such as the UV doses used. Furthermore, the source used for irradiation had a large bandwidth of 5 nm, which leads to noticeable broadening of the spectrum, and the extrapolation from 315 to 330 nm is questionable because there are no experimental data in this wavelength range.

**Fig. 10:**
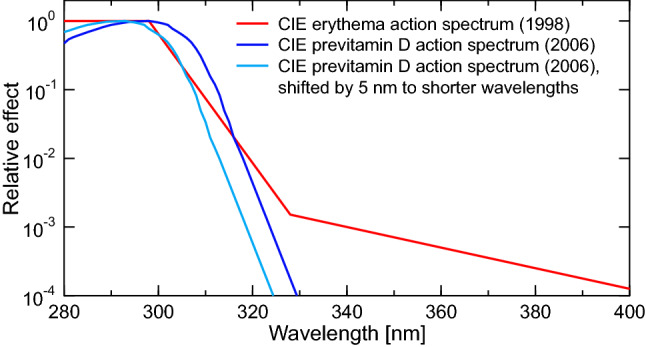
Comparison of CIE action spectra for erythema [10] and the cutaneous synthesis of previtamin D_3_ [365]. The effect of a 5-nm blue shift on the previtamin D_3_ action spectrum is also shown

Young et al. [367] have recently provided evidence that shifting the CIE action spectrum for previtamin D_3_ synthesis by 5 nm to shorter wavelengths (Fig. 10) would produce a more realistic action spectrum for the production of previtamin D_3_ in human skin. They exposed 75 volunteers to five lamp spectra with different spectral composition, and correlated the observed increase in serum 25(OH)D levels (the form of vitamin D used to assess vitamin D status) with the effective UV irradiance, UV_BE_. The action spectrum for calculating UV_BE_ was either the CIE spectrum in its unaltered form or a variant shifted in wavelength. The shift by 5 nm is plausible because the absorption spectrum of 7-dehydrocholesterol is also found to be shifted by about 5 nm to shorter wavelengths relative to the CIE action spectrum, even after adjusting for the spectral transmission of the skin’s outermost layer, the *stratum corneum* [366]. Furthermore, results obtained with the shifted action spectrum are consistent with calculations using alternative vitamin D action spectra proposed by Bolsée et al. [368], Olds [369], and van Dijk et al. [370], which are also shifted to shorter wavelengths relative to the CIE spectrum. These results suggest that the CIE standard [365] may need revision. However, the spectral change of solar spectra observed on the Earth (e.g., the difference between summer at the equator and winter in the Northern Hemisphere) is smaller than the difference in the spectral composition of the various artificial light sources used in the new experiment. The effect of the shift is, therefore, less important for natural sunlight, leading to the conclusion by Young et al. [367] that the CIE action spectrum (with no shift) remains adequate for risk–benefit calculations and the development of recommendations for healthy solar exposure. Along the same line, a recent assessment [371] concluded that the current CIE action spectrum [365] probably needs to be amended, but that it is acceptable to continue using this action spectrum for risk-benefits assessments until that work is completed.

An action spectrum for the inhibition of SARS-CoV-2 (the virus responsible for the COVID-19 disease) was recently measured. This spectrum is discussed by Bernhard et al. [2].

## Gaps in knowledge

Our assessment identified the following gaps in knowledge:

- Most ODSs are also GHGs and have a large effect on global warming. However, since ozone is also a GHG, depletion of ozone caused by ODSs has a cooling effect (Sect. 4.2). The net effect on temperatures at the Earth’s surface resulting from the direct (warming) effect of ODSs and the indirect (cooling) effect from ozone depletion induced by ODSs is uncertain because climate models disagree on the magnitude of the latter effect. While the balance of all studies suggests that the Montreal Protocol is highly effective in limiting temperature rise at the Earth’s surface, the magnitude of the effect remains uncertain.
- The effect of Antarctic ozone depletion on changes in sea ice surrounding Antarctica is not well understood.
- The effect of the Antarctic ozone hole on summertime weather in the Southern Hemisphere is uncertain. In particular, it is difficult to quantify if changes in weather are more affected by the year-to-year variability of the polar vortex, which is partly driven by changes in sea surface temperature of the Southern Ocean, or by the actual depletion of ozone within the vortex. It is also not clear how the coupling between the stratosphere and troposphere in weak vortex conditions will evolve under ozone recovery.
- While several studies have identified correlations between Arctic ozone changes and weather in the Northern Hemisphere, knowledge on how these linkages are mediated is incomplete.
- The paucity of measurements of the properties of aerosols in the UV-B range hampers our ability to accurately assess the effects of aerosols on a global scale as well as for urban regions. While efforts to improve this situation are underway—for example, EUBREWNET has recently started to provide AOD in the wavelength range from 306 to 320 nm (Sect. 6.1)—aerosol data in the UV-B range are currently available only for a few locations.
- Atmospheric blocking systems (stagnant high- or low-pressure synoptic systems) can cause week-long anomalies of UV radiation. It is not well understood how climate change may alter the frequency, persistence, and geographical extent and location of these blocking patterns, and their effect on UV radiation.
- One of the largest uncertainties in projecting changes to ozone and UV radiation during the twenty-first century is the evolution of GHG trajectories, which mostly depend on policy decisions and societal behavior.
- Uncertainties in projections of UV radiation arising from incomplete knowledge of future changes in aerosol and cloud optical properties are significant.
- The number of stations with high-quality spectral UV measurements has been declining during the last decade and the funding for many of the remaining stations is uncertain. If this trend continues, the scientific community may lose the ability to assess changes of UV radiation at the Earth’s surface and associated impacts, in order to verify new satellite UV data products with ground-based observations and to validate model projections.

## Conclusions

Virtually all studies published during the last four years confirmed that changes in UV radiation (typically assessed with the UVI) during the last 25 years have been small: less than 4% per decade for the UVI at the majority of ground stations, increasing at some sites and decreasing at others. Changes in the UVI outside the polar regions over the last 2–3 decades were mainly governed by variations in clouds, aerosols, and surface reflectivity (for snow- or ice-covered areas), while changes in TCO are less important. Variability in the UVI in Antarctica continued to be very large. In spring 2019, the UVI was at the minimum of the historical (1991–2018) range at the South Pole, while near record-high values were observed in spring 2020 and 2021, which were up to 80% above the historical mean. In the Arctic, some of the highest UV-B irradiances on record were measured in March and April 2020. For example in March 2020, the monthly average UVI over the Canadian Arctic was up to 70% higher than the historical (2005–2019) average, often exceeding this mean by three standard deviations.

Without the Montreal Protocol, the UVI at northern and southern latitudes of less than 50° would have increased by 10–20% between 1996 and 2020. For southern latitudes exceeding 50°, the UVI would have surged by between 25% (year-round at the southern tip of South America) and more than 100% (South Pole in spring).

Under the presumption that all countries will adhere to the Montreal Protocol in the future and that atmospheric aerosol concentrations remain constant, the UVI at mid-latitudes (30–60°) is projected to decrease between 2015 and 2090 by 2–5% in the north and by 4–6% in the south due to recovering ozone. Changes projected for the tropics are smaller than 3%.

Since most substances controlled by the Montreal Protocol are also greenhouse gases, the phase-out of these substances may have avoided warming by 0.5 to 1.0 °C over mid-latitude regions of the continents, and by more than 1.0 °C in the Arctic. ODSs contributed one-half of the forced Arctic sea ice loss in the latter half of the twentieth century. The uncertainty of changes in temperature and sea ice simulated by these models is still large.

Assessing the Montreal Protocol’s impact on solar UV radiation and climate, and their interaction, is impeded by several gaps in knowledge. The net temperature change at the Earth’s surface resulting from the direct (warming) effect of ODSs and the indirect (cooling) effect from ozone depletion is uncertain, because climate models disagree on the magnitude of the latter effect. While all studies support the role of the Montreal Protocol in limiting global warming, the magnitude of increases in temperatures that were averted remains uncertain. There is evidence that in both hemispheres polar ozone depletion in spring has an influence on weather; however, the mechanisms and magnitude of the effect are not fully understood. The lack of measurements of absorption properties of aerosols in the UV-B range hinders the assessment of the aerosols’ impact on UV-B radiation. One of the largest uncertainties in projecting changes in UV radiation during the twenty-first century is the incomplete knowledge of how GHGs will increase over time. Uncertainties in UV projections arising from inadequate understanding of future changes in aerosols and clouds are also significant.

Our assessment addresses several United Nations Sustainable Development Goals (SDGs) and their targets (https://sdgs.un.org/goals). Owing to the Montreal Protocol, large increases in UV-B radiation have been avoided and global warming reduced. By assessing how ozone depletion affects climate change, we contribute to SDGs 13.1 (“strengthen resilience to climate-related hazards and disasters”) and 13.2 (“integrate climate change measures into policy, strategy and planning”). Furthermore, by providing up-to-date information on the interactive effects of ozone depletion on UV radiation and climate, both in this assessment and the companion document titled “Questions and Answers about the effects of the depletion of the ozone layer on humans and the environment”, we address SDGs 13.3 (“improve education on climate-change mitigation”) and 17.14 (“enhance policy coherence for sustainable development”).

## Data Availability

All data generated or analyzed are either included in this published article or part of the analyses of papers cited**.**

## Footnotes

1 See the *Photochem. Photobiol. Sci*. themed issue entitled: Environmental effects of stratospheric ozone depletion, UV radiation, and interactions with climate change: UNEP Environmental Effects Assessment Panel, Quadrennial Assessment 2022 (10.1007/s43630-023-00374-9).

2 If not stated otherwise, the latitude ranges for both the Northern and Southern Hemispheres are defined as: polar latitudes (80°–90°); high-latitudes (60°–80°); mid-latitudes (30°–60°); low latitudes or tropics (0°–30°).

3 The Montreal Protocol was adopted in 1987 and was implemented in 1989 when it entered into force.

4 Total column ozone or TCO is the amount of ozone in a vertical column extending from the Earth’s surface to the top of the atmosphere. TCO is reported in Dobson Units or DU. One DU corresponds to a hypothetical layer of pure ozone with a thickness of 0.01 mm that would ensue if all ozone molecules in the vertical column were compressed to standard pressure (1013.25 hPa) and temperature (273.15 K or 0 °C). One DU corresponds to 2.69 × 10^16^ molecules per square centimeter of area at the base of this column. Averaged over the Earth’s surface, the TCO is about 300 DU, which relates to a layer of pure ozone that is three millimeters thick.

5 Irradiance is the radiant power (or radiant flux) received by a surface per unit area. “Radiant” indicates that the energy is received as electromagnetic radiation, and the surface is assumed horizontal unless otherwise specified.

6 The UV Index is calculated by weighting solar UV spectra with the action spectrum of erythema [10] and multiplying the result with 40 m^2^/ W. See also Sect. 11.

7 Large-scale perturbations in atmospheric circulation, typically manifesting as meandering of the jet stream.

8 Heterogeneous chemical reactions are chemical reactions between substances of different phases, e.g., gaseous, liquid, solid.

9 Representative Concentration Pathways are greenhouse gas concentration (not emission) trajectories adopted by the Intergovernmental Panel on Climate Change (IPCC) for its fifth Assessment Report. The pathways are used for climate modeling and research. They describe four climate futures, which differ in the amount of greenhouse gases that are emitted in years to come. The four RCPs, RCP 2.6, RCP 4.5, RCP 6, and RCP 8.5, are named after a possible range of radiative forcing values in the year 2100 relative to pre-industrial values (+ 2.6, + 4.5, + 6.0, and + 8.5 W m^−2^, respectively).

10 Coupled Model Intercomparison Project Phase 6.

11 Shared socio-economic pathway (SSP) scenarios describe a range of plausible trends in the evolution of society over the twenty-first century and were adopted by the Intergovernmental Panel on Climate Change (IPCC) for its sixth Assessment Report. The pathways are used for climate modeling and research, as different socio-economic developments and political environments will lead to different GHG emissions and concentrations. They describe five climate futures (SSP1–SSP5) that are combined with assumed amounts of greenhouse gases that are emitted in years to come. The CMIP6 simulations are based on seven SSPs (SSP1-1.9, SSP1-2.6, SSP2-4.5, SSP3-7.0, SSP4-3.4, SSP4-6.0, and SSP5-8.5), which are named after a possible range of radiative forcing (see footnote 14) values in the year 2100 relative to pre-industrial values (1.9, 2.6, 4.5, 7.0, 3.4, 6.0, and 8.5 W m^−2^, respectively), and have some equivalence to the “Representative Concentration Pathways” or RCPs used in IPCC’s fifth Assessment Report.

12 Goddard Earth Observing System Chemistry–Climate Model.

13 REF-C2 simulation of the NIWA-UKCA model (Implementation of the United Kingdom Chemistry and Aerosols (UKCA) model by New Zealand’s National Institute of Water & Atmospheric Research (NIWA)) [58] with exponentially increasing concentrations of ODSs at 3% per year added from 1974 onwards.

14 Radiative forcing quantifies the change in Earth’s energy balance (in W m^−2^) between incoming short-wave solar radiation and outgoing long-wave (thermal) IR radiation, either at the tropopause or at the top of atmosphere. If radiative forcing is positive at the tropopause, the temperature of the troposphere will increase.

15 Emergent constraints are physically explainable empirical relationships between characteristics of the current climate and long-term climate prediction that emerge in large ensembles of climate model simulations.

16 “World Avoided” scenarios such as the scenario discussed here are inevitably only estimates based on the state of current knowledge. They cannot consider possible changes in human behavior and policies that may come about when large changes in UV irradiance and their consequences would have become more obvious in the future. Nevertheless, these projections allow us to put the crucial benefits that the Montreal Protocol has brought to date into perspective.

17 The SAM is the leading mode of Southern Hemisphere extratropical climate variability describing a seesaw of atmospheric mass between the mid- and high-latitudes, with corresponding impacts on the strength of the circumpolar westerly winds. A positive SAM index corresponds to a poleward shift of the maximum wind speed, which results in weaker-than-normal westerly winds in the southern mid-latitudes.

18 The term “westerly jet” refers in the context to the maximum of westerly winds (i.e., winds blowing from west to east) close to the surface, not the jet stream in the upper troposphere. The jet’s latitude is defined as the latitude with the largest wind speed.

19 Long-wave radiation is electromagnetic radiation with wavelengths between 3 and 100 μm that is emitted from the Earth and its atmosphere in the form of thermal radiation. Long-wave radiation contrasts with short-wave radiation with wavelengths between ~ 0.3 and ~ 3 μm originating from the Sun.

20 Albedo is the proportion of the incident radiation that is reflected by a surface. Short-wave albedo refers to the fraction of the total incident solar irradiance in the wavelength range of ~ 0.3–3 μm that is reflected by the Earth’s surface. Albedo may also refer to the reflectivity in a certain wavelength range, such as the UV range.

21 The Arctic Oscillation (AO) or Northern Annular Mode (NAM) is analogous to the Southern Annular Mode (SAM) and characterizes the pattern of winds circulating around the Arctic. When the AO is in its positive phase, a ring of strong winds circulating the North Pole acts to confine colder air in the polar regions.

22 The position of the Sun in the sky is typically either described by the solar elevation, which is counted from the horizon, or the solar zenith angle (SZA), which is counted from the zenith (the imaginary point directly above a particular location). The solar elevation can be calculated as 90° – SZA.

23 The AOD is the sum of the aerosol scattering optical depth τ_sca_ and the absorption optical depth τ_abs_, which quantifies the attenuation of the direct solar beam due to scattering and absorption of photons: τ = τ_sca_ + τ_abs_. Instead of specifying τ and τ_abs_, the single scattering albedo (SSA) is often reported instead: SSA = τ_sca_ / (τ_sca_ + τ_abs_) = τ_sca_ / τ, resulting in τ_abs_ = (1 − SSA) × τ. A decrease in SSA, therefore, corresponds to an increase in absorption of radiation.

24 The global number of deaths from air pollution (particulate matter and gases such as tropospheric ozone and nitrous oxides) has been estimated at 4.2 million per year [5]. In comparison, the number of deaths from skin cancer was about 120,000 in 2020 (https://gco.iarc.fr/today/fact-sheets-cancers, accessed 13 November 2022).

25 Biomass burning is the burning of living and dead vegetation. It generally includes the human-initiated burning of vegetation for land clearing and land-use change as well as natural, lightning-induced fires.

26 The standard erythemal dose (SED) is a measure of cumulative erythemal UV radiation [177]. One SED is equivalent to an erythemally effective radiant exposure of 100 J m^−2^. Two SED may lead to erythema in individuals with freckled pale skin (Skin Type I, defined by the Fitzpatrick scale [178]). Longer exposure times are required for individuals with darker skin [179].

27 Aerosol Comparisons between Observations and Models (https://aerocom.met.no/).

28 Copernicus Atmosphere Monitoring Service (https://atmosphere.copernicus.eu/).

29 In meteorology, synoptic scale refers to a high- or low-pressure area with a horizontal length scale of the order of 1000 km or more.

30 Energetic particles considered here are highly energetic electrons, protons, neutrons, and ions that are accelerated into the atmosphere through various heliophysical and geomagnetic processes. They enter the atmosphere mainly in the geomagnetic polar regions (https://lasp.colorado.edu/home/mag/research/energetic-particle-precipitation).

31 Grand solar minima are defined as periods when several solar cycles exhibit lesser than average activity for decades or centuries.

32 Total Solar Irradiance (TSI) is the solar radiative power per area integrated over all wavelengths that is incident on the Earth’s upper atmosphere.

33 The study did not include the chemical impact of halogen compounds, as there is no reliable information on their emissions from Toba. The modeled effect on ozone mainly occurs because absorption by SO_2_ and scattering by the aerosol layer reduces the flux of solar UV﻿-C radiation reaching the lower stratosphere. Solar UV-C radiation with wavelengths shorter than 242 nm initiates the formation of ozone in the stratosphere because it leads to the photolysis of oxygen molecules (O_2_). The resulting oxygen atoms react with O_2_ to form ozone (O_3_). Less UV-C flux below the aerosol layer therefore leads to less ozone production. If ozone loss by halogen compounds had been included also, the modeled ozone decline and increase in UV-B radiation at the Earth’s surface would have been even larger.

34 INM-RSHU: Institute of Numerical Mathematics – Russian State Hydrometeorological University; ERA-Interim: European Centre for Medium-Range Weather Forecasts Re-Analysis; TOMS: Total Ozone Mapping Spectrometer.

35 ERA-Interim is a global atmospheric reanalysis published by the European Centre for Medium-Range Weather Forecasts (ECMWF, https://www.ecmwf.int/) that is available from 1 January 1979 to 31 August 2019.

36 EMAC CCM is the European Centre for Medium-Range Weather Forecasts–Hamburg (ECHAM)/Modular Earth Submodel System (MESSy) Atmospheric Chemistry Model.

37 (2Z,6Z)-2,6-bis(2-(2,6-diphenyl-4H-thiopyran-4-ylidene)ethylidene)cyclohexanone.
